# Progesterone Actions and Resistance in Gynecological Disorders

**DOI:** 10.3390/cells11040647

**Published:** 2022-02-13

**Authors:** James A. MacLean, Kanako Hayashi

**Affiliations:** Center for Reproductive Biology, School of Molecular Biosciences, Washington State University, 1770 NE Stadium Way, Pullman, WA 99164, USA

**Keywords:** progesterone, progesterone resistance, endometrium, endometriosis, adenomyosis, PCOS

## Abstract

Estrogen and progesterone and their signaling mechanisms are tightly regulated to maintain a normal menstrual cycle and to support a successful pregnancy. The imbalance of estrogen and progesterone disrupts their complex regulatory mechanisms, leading to estrogen dominance and progesterone resistance. Gynecological diseases are heavily associated with dysregulated steroid hormones and can induce chronic pelvic pain, dysmenorrhea, dyspareunia, heavy bleeding, and infertility, which substantially impact the quality of women’s lives. Because the menstrual cycle repeatably occurs during reproductive ages with dynamic changes and remodeling of reproductive-related tissues, these alterations can accumulate and induce chronic and recurrent conditions. This review focuses on faulty progesterone signaling mechanisms and cellular responses to progesterone in endometriosis, adenomyosis, leiomyoma (uterine fibroids), polycystic ovary syndrome (PCOS), and endometrial hyperplasia. We also summarize the association with gene mutations and steroid hormone regulation in disease progression as well as current hormonal therapies and the clinical consequences of progesterone resistance.

## 1. Introduction

Progesterone is one of the key steroid hormones in the complex regulation of female reproductive functions, largely controlled in multiple organs such as the uterus, ovary, mammary gland, and brain (reviewed in [1]). High levels of progesterone are produced from the corpus luteum and placenta, which are necessary to maintain a successful pregnancy. Beyond pregnancy, the majority of female reproductive processes are also controlled by progesterone, including oocyte maturation, ovulation, menstruation, facilitation of implantation and decidualization, uterine growth, suppression of myometrial contraction, mammary gland development, regulation of milk production, and sexual behavior as summarized in [1]. Progesterone actions are mediated by progesterone receptors (PGR). Female mice with ablation of *Pgr* (PGRKO) clearly show complete sterility with multiple abnormalities in reproductive functions: ovulation failure, hyperplastic uterine response to estrogen and progesterone, decidualization failure, disruption of mammary gland development, and a lack of sexual behavior [2]. PGR consists primarily of two nuclear isoforms (PRA and PRB) which have distinct expression patterns and functional profiles [3]. A single gene with independently regulated promoters is responsible for encoding PRA and PRB, producing similar proteins with PRB having an extra 164 amino acids at the N-terminus [4]. Characterization of PRA or PRB mutations in female mice indicates that PRA mainly regulates uterine PGR functions, whereas PRB is important for mammary gland development [5,6]. While progesterone and its activation of downstream mechanisms are mediated by PGR, the actions of progesterone are tightly integrated with estrogen and its responsive signaling pathways [1,7,8,9].

**Figure 1:**
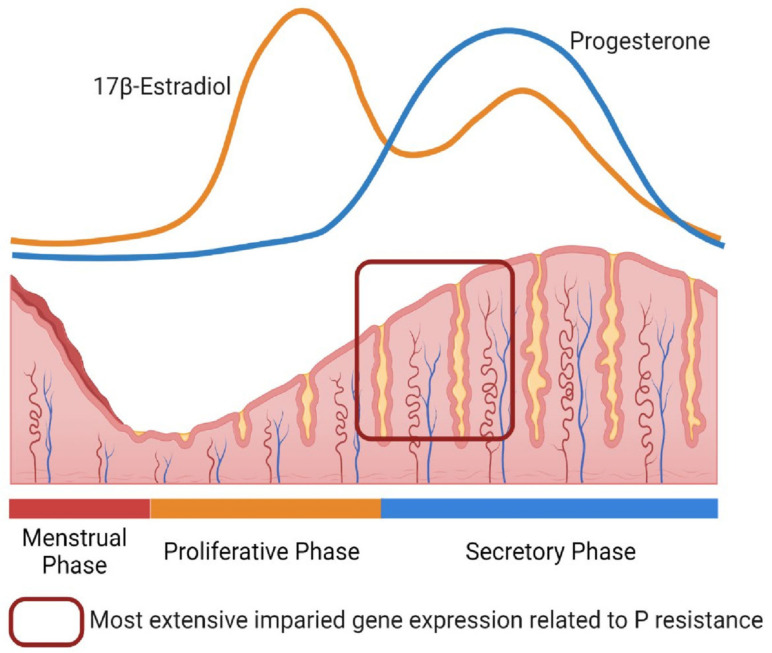
Menstrual cycle. Steroid hormone-derived endometrial changes. Created with BioRender.com.

The endometrium, the lining of the uterus, is one of the most dynamic tissues in adults. The endometrium undergoes a persistent cycle of remodeling including shedding, proliferation to regenerate layers, and differentiation during menstruation. These processes are mainly under the control of ovarian-derived steroid hormones, estrogen and progesterone (Figure 1). In the human endometrium, estrogen drives tissue repair and epithelial proliferation during the proliferative phase and estrogen and progesterone promote thickening of the endometrium following ovulation. Increased progesterone concentrations eventually inhibit estrogen action to induce decidualization during the secretory phase [10,11]. During the reproductive years in women, which span several decades, endometrial layers repeatably repair, proliferate, and then shed to maintain active reproductive functions. However, the loss of hormone balance, disruption of hormone-dependent downstream signaling mechanisms, and/or aberrant inflammation cause hormone insensitivity, estrogen dependence/dominance, and progesterone resistance. A decreased cellular responsiveness to progesterone and/or a failure to activate PGR leads to compromised implantation and the development of gynecological diseases [12,13,14,15]. Based on the tightly regulated functions of estrogen and progesterone in the endometrium, controlling estrogen and progesterone levels by hormonal therapies has been the primary strategy to manage endometrial physiology and menstruation. However, the endometrium is sensitive to environmental cues (including endocrine-disrupting exposure), inflammatory signals, and other unknown factors which alter and dysregulate hormone-derived endometrial cellular functions. These lead to changes in downstream gene expression and epigenetic marks which further complicate endometrial tissue regulation by establishing a hormone-insensitive environment [12,16,17,18,19].

The goal of this review is to highlight the inroads by which faulty progesterone signaling mechanisms and cellular responses to progesterone lead to the initiation or progression of gynecological disorders, including endometriosis, adenomyosis, leiomyoma (uterine fibroids), polycystic ovary syndrome (PCOS), and endometrial hyperplasia. We focus on summarizing the established and putative gene mutations and misregulation of steroid hormone signaling in disease progression, as well as current hormonal therapies and the clinical consequences of progesterone resistance (Table 1).

## 2. Progesterone Resistance in the Endometrium

Progesterone resistance is widely recognized as endometrial progesterone unresponsiveness with subsequent dysregulation of epithelial and stromal gene networks in the endometrium [12,13,14,15]. The accumulation of impaired progesterone and persistent estrogen actions from one menstrual cycle to another induces abnormal pathophysiological changes in the endometrium. These could lead to the development of endometrial-related disorders such as endometriosis, adenomyosis, PCOS, endometrial hyperplasia, and implantation failure [12,13,14,15,20]. To date, large-scale gene expression studies in endometriosis [16,17] and PCOS [21] have revealed that progesterone-regulated genes are most likely altered in their endometrial expression during the early- and mid-secretory phases and these dysregulated genes are associated with loss of normal endometrial functions and disease establishment and progression (Figure 1). Aberrant induction of inflammatory and/or oncogenic related genes are also strongly correlated to the dysregulation of endometrial functions in these diseases [12,22,23]. Epigenetic alterations, including hypermethylation which reduces PGR expression [14,24] and the expression of other endometrial genes directly or indirectly linked to progesterone [25,26,27,28,29], can result in progesterone unresponsiveness. The recent advances in whole-exome or genome sequencing (WES or WGS) have allowed the identification of common somatic mutations in the endometrial epithelial cells that potentially lead to the development of endometriosis and/or adenomyosis [30,31,32,33,34]. Especially significant are *KRAS* mutations which are observed in adenomyosis co-occurring endometriosis with downregulated PGR expression [31]. Aberrancies of gene expression, epigenetic marks, and/or gene mutations likely influence progesterone signaling in the endometrium. Further details of the mechanisms in each disease are summarized in the sections below.

## 3. Endometriosis

### 3.1. Disease Features in Endometriosis

Endometriosis is defined as the presence of endometrial-like tissues containing endometrial glands and stroma, often with hemosiderin outside of the uterus, mainly on pelvic organs and tissues [35,36,37,38]. Retrograde menstruation, which is the reflux of menstrual debris containing endometrial tissues through the fallopian tubes into the pelvic cavity [39], has been widely accepted as the origin of endometriotic tissues. However, as retrograde menstruation occurs in more than 90% of menstruating women [40], other factors must contribute to the establishment of endometriotic lesions [35,37,38]. The common types in endometriosis include superficial peritoneal lesions, deep-infiltrating endometriosis, and ovarian endometrioma, as well as extensive adhesion to the lesions with other pelvic organs which is also one of the common features of endometriosis [35,36,37,38]. The prevalence of endometriosis is estimated to affect approximately 10% of reproductive-age women, representing nearly 190 million women worldwide [37,41]. It is associated with debilitating chronic pelvic pain and infertility which substantially reduce the quality of life of women and their families [38,42]. Indeed, the health care costs for endometriosis in 2008 have been estimated at approximately $4000 per person in the United States (U.S.) [37,43].

### 3.2. Current Treatments in Endometriosis

Because endometriosis is an estrogen-dependent disorder, current treatments focus on suppression and/or inhibition of local or systemic estrogen production and its actions. However, medical treatments such as hormonal therapies do not remove lesions and therefore cannot completely abrogate disease-associated symptoms [44,45]. The most common and long-term treatment is the use of oral contraceptive pills formulated with estrogen and progestin or progestin-only. These are effective at reducing chronic pelvic pain and/or dysmenorrhea [46,47]. The consensus from clinical reports in the literature has shown that contraceptive pills are beneficial in women with dysmenorrhea and endometriosis-associated pain [44,45]. The levonorgestrel intrauterine device (LNG-IUD) often results in amenorrhea, which is especially effective for dysmenorrhea [48]. However, the efficacy of progestin therapy is limited due to only minimally improving the symptoms in the short term, and symptoms frequently reappear after treatment discontinuation [44,49]. GnRH agonists are preferentially used for the second-line pharmacological treatment of endometriosis-associated pain and act to inhibit estrogen production by the ovary, limiting its cellular functions [35,50]. While GnRH agonists effectively reduce the deleterious effects of endometriosis and lesion development, they are not compatible with the restoration of fertility as folliculogenesis in the ovary is shut down. Laparoscopic surgery to remove lesions can provide some pain relief. However, hormonal treatments, as well as laparoscopic surgeries, are often of limited efficacy with high recurrence rates, frequent side effects, additional costs, and potential morbidity [51]. The recurrence rate of surgical excision of lesions is over 50% after five years [52,53]. GnRH agonist therapy induces temporary menopause with unwanted side effects and over 50% of women exhibit a recurrence of symptoms within two years [54,55]. Progesterone resistance is a major complication for progestin therapy, leading to the escalation of estrogen activity [8]. Thus, a critical need exists to develop more effective therapies for endometriosis that target the biologically important mechanisms that underpin the pathophysiology of this disease.

### 3.3. PGR Expression in Endometriosis

PGR mediates progesterone actions in the progesterone responsive tissues, including the endometrium. While PRA and PRB isoforms are detected in human eutopic endometrium with endometriosis during the menstrual cycle, both the expression of PRA and PRB are generally lower in any type of ectopic lesions [56]. In particular, deficient PRB expression has been reported from several groups [27,57,58]. The consensus abnormal regulatory mechanism stems from epigenetic alteration of the *PGR* promoter regions in the chronic high cytokine state induced by the presence of lesions. This results in differential expression at the *PRB* transcription start sites that are hypermethylated, but the regions associated with *PRA* transcription are not, leading to the disproportional expression of the two PGR isoforms [26,27,59]. However, the relative abundance of PGR isoforms in the cells cannot explain the progesterone actions due to the complex mechanisms of PGR signaling [12,60]. For instance, endometrial stromal cells become more sensitive to PGR signaling during the decidualization process when the cyclic AMP (cAMP) levels are elevated and then the protein kinase A (PKA) pathway is activated [12,61,62]. Furthermore, some studies have reported no differences of PGR isoforms observed in the eutopic endometrium with/without endometriosis [63,64]. Ectopic rectosigmoid lesions do not show any different PGR expressions either [65]. Those results suggest that it would be hard to conclude that loss or altered PGR expression alone in the eutopic endometrium or the ectopic lesions directly leads to progesterone resistance in endometriosis.

### 3.4. Altered Gene Expression in Endometriosis

The pathogenesis of endometriosis is a complex process and still remains to be fully understood. Endometrial stem or progenitor cells have been proposed to contribute to the development of endometriosis [66]. Altered local immune dysfunction and inflammatory responses can be the consequence of endometriosis [67]. Elevated ESR2 activity in retrograded endometrial tissues interacts with the cytoplasmic inflammasome to increase IL-1β to enhance cellular adhesion and proliferation, as well as epithelial-mesenchymal transition (EMT), leading to an escape from immune surveillance and development of endometriotic lesions [68]. On the other hand, estrogen is a key promoter for endometriotic lesion growth and progression and establishment of the chronic endometriotic environment within the pelvic cavity, whereas progesterone is a master regulator tightly controlling estrogen actions. Endometriosis and its established inflammatory environment disrupt the balance of hormonal regulation and reduce coordinated progesterone responses or vice versa, resulting in the development of progesterone resistance [12,13,14,69].

Progesterone resistance includes the disruption of progesterone-dependent genes in the eutopic endometrium with endometriosis [16,17,70,71]. Burney et al. [16] have reported impaired gene expression in the eutopic endometrium with endometriosis throughout the cycle, whereas the most extensive changes are incomplete transitions of the endometrium occurring from the proliferative to early secretory phase, especially in the persistence of estrogen-regulated genes. The best characterized dysregulated genes identified in the secretory phase are progesterone targets such as *FOXO1A*, *MIG6,* and *CYP26A1*, indicating the attenuation of progesterone response [16]. Garcia-Alonso et al. have recently profiled single-cell (sc) and temporal/spatial transcriptome of human endometrium using 10× scRNA-seq and single-nucleus RNA-seq, as well as Genomics Visium platforms [72]. Spatiotemporal characterization during the proliferative and secretory phases of endometrium shows that *SOX9+LGR5+* populations are present that are associated with proliferative and regenerative potential stem cell niche. When the authors explore the expression of specific epithelial genes from endometriosis public data sets, *SOX9+LGR5+* subset populations show upregulated epithelial gene markers (*WNT7A* and *KRT17*) in the lesions which are similar to those expression levels in proliferative endometrium [72]. Furthermore, WNT and NOTCH signaling regulate ciliated and secretory epithelial cells with opposing roles to distinct epithelial lineages. Although it is not clear whether the expression levels are compared with eutopic endometrial tissues from endometriosis and control patients, the studies using these new techniques in endometriosis will improve further understanding of altered gene regulation in specific cellular populations of endometriosis. Currently, two preprint papers are available for single-cell transcriptomics in endometriosis lesions [73,74].

### 3.5. Altered PGR Signaling in the Eutopic Endometrium with Endometriosis

Endometriosis is known as an estrogen-dependent disease because alterations of progesterone-regulated genes and signaling cause persistent estrogen activation [12]. To modulate PGR downstream target genes, steroid receptor coactivators (SRC) are first recruited for the modification of chromatin structures to form complexes with PGR and other transcription factors [75,76]. Based on a series of studies from O’Malley’s group [77,78,79,80,81,82], SRC-1 and SRC-2 are likely most relevant to regulating progesterone-dependent endometrial functions. On the other hand, SRC functions also contribute to PGR-dependent disease progression. Loss of *Src-1* reduces endometriotic lesion progression using a mouse model of endometriosis [83]. A newly identified 70 kDa SRC-1 isoform is highly elevated in the ectopic lesions; this lesion-specific form is generated by abnormal TNFα-induced MMP9 activity and it prevents TNFα-mediated cell death induced by estrogen-dependent full-length SRC-1 function. These studies suggest that the endometriosis-induced inflammatory environment alters the molecular properties of SRC-1, and the SRC-1 isoform promotes endometriosis progression via EMT and epithelial cell invasion [83].

Dysregulation of PGR signaling as a part of progesterone resistance has been reported in endometriosis [9,12,13,14]. IHH signaling is one of the major PGR-mediated pathways which inhibits the epithelial proliferation stimulated by estrogen that is required for successful implantation, followed by stimulation of COUP-TFII and BMP2 for successful decidualization [84,85,86,87]. Nuclear and cytoplasmic IHH in the endometrium generally increases from the late proliferative to secretory phases [88]. However, both nuclear and stromal IHH expression decreases in the secretory phase of eutopic endometrium with endometriosis [88]. The loss of *IHH* in the mouse uterus produces a similar phenotype to PGRKO mice with global ablation of PGR signaling, confirming the importance of IHH signaling in the endometrium [86]. The expression of COUP-TFII is also reduced in the eutopic endometrium with endometriosis and ectopic lesions [89]. Other critical PGR signaling mediators: WNT4 [90], HOXA10 [71], MIG6 [16,91,92], FOXO1 [16,93], and CRISPLD2 [94] decrease in the eutopic endometrium with endometriosis, especially during the secretory phase. WNT4 [95], HOXA10 [96,97], MIG6 [91,92], and FOXO1 [98,99] are PGR targets and/or mediators in the endometrium and critical for embryo implantation and decidualization. These results indicate that dysregulated and/or disrupted PGR targets in the eutopic endometrium can cause unresponsiveness of progesterone actions, probably further leading to disease progression.

NOTCH signaling modulates crucial mechanisms for endometrial decidualization and successful pregnancy [100,101]. NOTCH signaling receptors, ligands, and direct target genes are also decreased in the eutopic endometrium of women and baboons with endometriosis [93]. Knockdown of NOTCH1 disturbs decidualization with the downregulation of FOXO1 in vitro stromal cell culture [93]. On the other hand, NOTCH intracellular domain 1, NICD1, is increased and inversely associated with its expression with decreased PGR expression in the eutopic endometrium and ectopic lesions [102]. Furthermore, inhibition of NOTCH signaling activation restores progesterone responsiveness and PGR expression [102].

Progesterone resistance can be explained by the dysregulation of immunophilin FKBP52, a PGR chaperone protein that governs progesterone actions like implantation and decidualization in the uterus [103,104]. In endometriosis patients, FKBP52 is reduced in both the proliferative and secretory phases within eutopic endometrium and ectopic lesions [105,106]. Deletion of FKBP52 enhances endometriotic lesion growth and progression with increased inflammation, proliferation, and angiogenesis [105]. In vitro cell culture studies suggest that FKBP52 expression might be regulated by HOXA10 [106] or miR-29c [107]. Reduction of FKBP52 is also observed in a non-human primate model of endometriosis [108].

The presence of altered chronic inflammation is one of the well-known features of endometriosis [37,38]. Activated STAT3 and NFκB signaling by cytokines and chemokines associated with immune cells have been shown in endometriosis [109,110,111,112,113]. Aberrant p-STAT3, co-occurring with HIF1A, presents in the eutopic endometrium in humans and non-human primates with endometriosis [111]. Although STAT3 directly interacts with the PRA isoform and is required for normal endometrial functions such as decidualization [114], STAT3 constitutive stimulation is activated by inflammatory factors from tissue residential or peritoneal immune cells in endometriosis [112,113,115]. Yoo et al. [116] have reported that PIAS3, which is a negative regulator of STAT3 activity, is decreased in the eutopic endometrium with endometriosis in humans and non-human primates inversely associated with aberrant p-STAT3. In support of PIAS3 having an active role in preventing endometriosis, an in vitro study has shown that INFγ can reduce PIAS3 but increases p-STAT3, suggesting aberrant STAT3 activation by attenuation of PIAS3 [116]. On the other hand, overexpression of KRAS and the histone deacetylase SIRT1 has been observed in the eutopic endometrium with endometriosis [117]. In the mouse model, *Kras* activation increases SIRT1 and decreases PGR target genes, including the genes related to IHH signaling [117]. SIRT1 colocalizes with BCL6, a known target of STAT3 [118], in the nuclei of eutopic endometrium [117,119]. BCL6 binds the *GLI1* promoter, a transcription factor mediating the Hedgehog pathway, to suppress *GLI1* transcription, resulting in the disruption of progesterone-mediated IHH signaling [117]. The *KRAS* gene is often mutated to become constitutively activated in the endometrium [30,31,33,34,120]. The involvement of somatic mutations in endometriosis is summarized in Section 3.7.

SOX17 has been identified as an essential uterine PGR-regulated gene [121] and regulates implantation and gland development, especially epithelial proliferation and differentiation through IHH signaling [122,123]. In endometriosis, SOX17 is decreased in the proliferative and secretory phases of eutopic endometrium compared with normal endometrium [124]. Reduced SOX17 is correlated with reduced IHH expression. Interestingly, transcriptome profiles with loss of *Sox17* overlap with those from *Arid1a* ablation [124]. Reduction of ARID1A is observed in the *Sox17*-ablated uterus, but SOX17 exhibits normal expression in the *Arid1a*-ablated uterus, indicating that ARID1A is potentially regulated by SOX17. Because ablation of *Arid1a* disrupts PGR signaling and ARID1A can directly bind to PRA, ARID1A is essential for normal endometrial functions and reduced ARID1A expression can alter PGR signaling leading to progesterone resistance in the endometrium with endometriosis [125]. Kim et al. [126] have also recently demonstrated that one of the histone deacetylases, HDAC3, has an essential role in endometrial decidualization and loss of *Hdac3* in the uterus disturbs PGR signaling. HDAC3 expression is decreased in the endometrium with endometriosis and might be involved in a part of progesterone resistance.

### 3.6. Epigenetic Alterations in Endometriosis

Dysregulated PGR target genes can be explained as consequences of altered methylation status in the endometrium [14,24]. A well-known PGR target, HOXA10, reduces its expression in the eutopic endometrium with endometriosis [71]. Wu et al. have first reported that reduction of *HOXA10* expression is due to hypermethylation of the putative promoter regions of *HOXA10* in the eutopic endometrium with endometriosis [29]. Kim et al. [127] have demonstrated that a baboon model of endometriosis results in a gradually decreased *HOXA10* in the eutopic endometrium after the lesion induction, owing to the increased DNA methylation of the proximal promoter of *HOXA10*. Hypermethylation of the *HOXA10* promoter region is also observed in the mouse uterus with induced endometriosis [128]. Aberrant promoter methylation of *SF1*, *PGRB*, *ESR2*, which are important for the response of progesterone, has also been reported in the eutopic endometrium and ectopic lesions with resulting progesterone resistance [25,26,27,28]. On the other hand, the Giudice group [18] has reported the analysis of global DNA methylation in the eutopic endometrium with or without endometriosis during the proliferative, early-, and mid-secretory phases show no differential methylation of specific promoter regions of the above genes. However, their results show a significant number of loci with altered DNA methylation that are found in the mid-secretory phase of the endometrium with endometriosis. Furthermore, alterations of DNA methylation are associated with altered gene expression related to endometrial function, including cell proliferation, inflammation, angiogenesis, and steroid hormone response. Thus, epigenetic modification in the eutopic endometrium contributes to disrupted hormonal actions causing progesterone resistance in endometriosis [18]. It is known that steroid hormones (estrogen and progesterone) and chronic inflammation can alter the chromatin landscape in the endometrium [12,18]. The Giudice group [19] has further examined the effects of 17β-estradiol, progesterone, and their combination on the DNA methylome and transcriptome, comparing eutopic endometrial stromal fibroblasts isolated from normal (disease-free) and endometriosis patients from different disease stages. 17β-estradiol and progesterone individually and together promote unique profiles in DNA methylome. Overall, 17β-estradiol alone induces broad changes in the DNA methylome in normal endometrium, but progesterone alone has a lesser effect. The combination of 17β-estradiol and progesterone results in reduced numbers of differentially methylated loci compared to 17β-estradiol alone. Hormonal responses to the DNA methylome in the eutopic endometrial stromal fibroblast are aberrant in the early- and late-stage endometriosis, which is probably due to already existing DNA methylation marks prior to hormone treatments. Additionally, hormone-induced methylation alterations are largely enriched within enhancers and intergenic regions but are minimally involved in proximal promoters and CpG islands.

Dyson et al. [129] have reported that members of the GATA transcription factor family are differentially methylated in stromal cells from normal endometrium without endometriosis and ectopic stromal cells isolated from ovarian endometrioma. GATA2 regulates genes necessary for decidualization in the normal endometrium. *GATA2* is hypermethylated in the ectopic stromal cells. In contrast, *GATA6* is normally hypermethylated and its expression restricted, but in lesions, it becomes hypomethylated and replaces *GATA2,* which promotes endometriotic phenotypes. This study did not examine the methylation statuses of *GATA2* or *GATA6* in the eutopic endometrium with endometriosis, including different stages of the disease and different cyclic phases. This analysis would be of interest as it would conclusively determine whether this switch is causative of the disease or a result of tissue differences after the establishment of endometriosis.

### 3.7. Genomic Alterations and Somatic Mutations in Endometriosis

Endometriosis is a heterogeneous and complex disease. Currently, there are no well-characterized markers and no distinct causative evidence of endometriosis risk variants that are associated with putative genes of interest [38,130]. Genome-wide association studies (GWAS) have identified loci of interest with or without endometriosis, whereas it remains unclear how genome-wide significant loci contribute to the endometriosis pathogenesis [37]. However, analysis of genes located nearest to the risk loci indicates the impact of cell adhesion, migration, angiogenesis, inflammation, and hormone-related pathways, specifically WNT, MAPK, and STAT3 signaling under different conditions such as stages, fat distribution, pain scales, and cancers [37,38].

On the other hand, WES analyses show somatic epithelial mutations in ovarian endometrioma, deep-infiltrating endometriosis, and normal endometrium [30,32,34]. Suda et al. [34] have identified a number of somatic mutations that are associated with ovarian cancer in laser captured epithelial cells from ovarian endometrioma and normal endometrium. *KRAS* and *PIK3CA* are found especially as the most common mutations in both endometrioma and endometrium. Anglesio et al. [30] have reported that epithelium from deep-infiltrating endometriotic lesions carries known cancer driver mutations: *KRAS*, *PPP2R1A*, *PIK3CA,* and *ARID1A* from 5 of 24 patients (21%). A recent study [31] has indicated that *KRAS* mutations are more frequent in cases of adenomyosis with co-occurring endometriosis, leading to a reduced efficacy of progestin therapy by the silencing of PGR, and can be a driver for adenomyosis development as described in further detail in the adenomyosis section below.

## 4. Adenomyosis

### 4.1. Disease Features in Adenomyosis

Adenomyosis is characterized by the infiltration of endometrial-like tissues composed of glands and stroma into the myometrium [131]. It produces a diffusely enlarged uterus with ectopic adenomyosis lesions surrounded by the hypertrophic myometrium [132,133]. Adenomyosis has been considered to result in an invagination of the endometrium from the breakdown of the junctional zone between basalis endometrium and myometrium [134,135,136]. On the other hand, it has been suggested that de novo generation of endometrial tissues in ectopic locations such as embryonic Mullerian stem cells and adult stem and/or progenitor cells is an alternative theory [135,137,138,139]. Although adenomyosis and endometriosis share several histological and molecular features [140], patients with adenomyosis often suffer menorrhagia and pregnancy loss, including recurrent implantation failures and miscarriages [141,142]. The prevalence of adenomyosis can be up to 20~35% based on data from patients who underwent hysterectomy [143,144,145] though there are large variations depending on the studies [146]. Because hysterectomy is the definitive cure for adenomyosis, a recent population-based study in the U.S. has reported that ~82% of patients among women aged 16–60 years following incident adenomyosis undergo hysterectomy [147]. The highest incidence of adenomyosis is in women in their 40’s, 41–45 years (27.3%), but 36–40 (20.7%) and 46–50 years (19.6%) also show higher prevalence [147], probably due to adenomyosis often being histologically diagnosed/confirmed after hysterectomy [148]. On the other hand, recent advanced imaging systems are able to detect the presence of smaller adenomyosis lesions. Indeed, transvaginal ultrasound (TVUS) or magnetic resonance imaging (MRI) have indicated >30% of women aged 18–30 years [149] or 60% of patients aged 18–42 years [150] might already have adenomyosis. Thus, initiation of adenomyosis, i.e., abnormal invagination of the endometrium into the myometrium, has occurred in younger reproductive-age women [148]. Therefore, the identification of marker genes or risk factors for early identification and treatment of adenomyosis needs to be developed.

### 4.2. Current Treatments in Adenomyosis

Adenomyosis is an estrogen-dependent gynecological disease like endometriosis. The progression of adenomyosis lesions with hypertrophic myometrium is enhanced by estrogen and abrogated by progesterone [136]. For those reasons, hormonal treatments such as GnRH agonists, progestins, and oral contraceptives have been used to suppress pain symptoms and abnormal heavy menorrhagia [132,151,152]. GnRH agonists are effective in causing systemic hypoestrogenism to reduce the size of adenomyosis and improve adenomyosis-associated symptoms [153,154,155], whereas the side effects of GnRH agonists (menopausal symptoms) limit their use. LNG-IUD has been considered most effective and successfully used to treat adenomyosis because it is efficient in suppressing menstrual bleeding [156,157,158,159,160]. The efficacy of LNG-IUD is generally higher than other progestin-based treatments [151,152]. This is because LNG-IUD consistently suppresses the menstrual cycle. Radzinsky et al. [161] have reported that LNG-IUD is also effective in reducing chronic pelvic pain. While LNG-IUD can be a cost-effective and reliable long-term treatment for adenomyosis patients, the efficacy of LNG-IUD significantly reduces in a large volume uterus [162].

### 4.3. KRAS Mutations and Progesterone Treatment in Adenomyosis

Owing to PGR expression being less intense in adenomyosis lesions [136], progesterone resistance and hyperestrogenism are always a concern when using progestin-based therapies [136,163]. In support of this, the PRB isoform is also known to be downregulated in the eutopic and ectopic endometrium in adenomyosis, especially with severe cases [164,165]. However, the mechanisms of impaired progesterone signaling in the eutopic and ectopic endometrium, where adenomyosis is present, are not fully understood. A study published by Inoue et al. [31] has indicated that *KRAS* mutations can be a driver of adenomyosis development and progression and linked to the PGR downregulation. This group performed the WES to understand the comprehensive genomic characterization of adenomyosis with co-occurring endometriosis and leiomyoma. Their WES detected 134 unique synonymous and non-synonymous single-nucleotide variations (SNV) in 31/51 (60.8%) adenomyosis cases, suggesting that adenomyosis is a clonal disorder with somatic mutations [31]. It was shown that a *KRAS* mutation at the location of G12, which is well-known as an oncogenic mutation, is the highest alteration (~37%) in adenomyosis. Additionally, isolation of epithelial cells by laser capture microdissection (LCM) reveals that the *KRAS* mutations have most likely occurred in the endometrial epithelial cells. Furthermore, the co-occurring gain of function mutations of *KRAS* with the *PIK3CA* p.H1047 is frequently observed in both adenomyosis and endometriosis. As an important finding, *KRAS* mutations are observed in not only adenomyosis lesions but also histologically normal endometrial tissues. The presence of *KRAS* and *PIK3CA* mutations in the normal endometrium is consistent with recent publications [33,34]. Thus, frequent *KRAS* mutations in the normal endometrium might initiate invasive and proliferative cellular functions to develop ectopic adenomyosis [31]. Importantly, *KRAS* mutations have been found to be significantly high in adenomyosis lesions from the patients who have been treated with dienogest (an oral progestin, 84%) but do not respond well and eventually underwent hysterectomy compared with the patients without dienogest (26%). The study further confirms less sensitivity to dienogest treatment in the *KRAS* mutated lesions, probably due to epigenetic silencing of PGR expression by *KRAS* mutations [31]. Therefore, the authors conclude that adenomyosis etiology is strongly associated with *KRAS* mutations in the endometrium, as well as the status of *KRAS* mutations is likely to be a critical factor in selecting effective medical treatments. Thus, the assessment of disease progression can be predicted by the frequency of *KRAS* mutations [31].

## 5. Leiomyoma (Uterine Fibroids)

### 5.1. Disease Features in Leiomyoma

Leiomyomas are benign tumors of smooth muscle cells and fibroblasts developed in the myometrium [166,167]. The excessive accumulation of extracellular matrix (ECM) with collagens is one of the distinguishing characteristics of leiomyoma [168]. The prevalence of leiomyoma varies among the studies (4.5–68.6%) depending on study populations, races, ages, and diagnostic methods [169]. The estimated cumulative incidence of leiomyoma by the age of 50 is >80% for African-American women and ~70% for Caucasians, suggesting that African-American women show a higher risk of developing uterine leiomyoma [170]. In addition, leiomyomas in African-American women generally develop tumors that are larger in size and cause more severe symptoms than those in women of other races [169,170]. Although the majority of women can remain asymptomatic, leiomyoma can cause severe and chronic symptoms; heavy, irregular, and prolonged menstrual bleeding with accompanying anemia, pelvic pain, and dysmenorrhea, as well as fertility issues and labor obstruction [167,171,172]. Treatment of leiomyoma depends on the severity of symptoms and whether patients desire to become pregnant and/or the sizes and locations of the leiomyoma. Although medical treatments are available to specifically control leiomyoma-associated symptoms, nearly 75% of leiomyoma patients end up having hysterectomy surgery in the U.S. [173], which represents the impetus for one-third to half of all hysterectomies [174,175,176]. Although leiomyoma is not a disease stemming from the endometrium, it is one of the gynecological diseases regulated by steroid hormones. The symptoms of diseases and treatment options are similar to other gynecological diseases.

### 5.2. Current Hormonal Treatments and Roles of Progesterone in Leiomyoma

The cause of leiomyoma-associated heavy bleeding has not been fully understood. Medical treatments primarily focus on reducing abnormal uterine bleeding. Because estrogen has been considered the primary driver to stimulate the progression of leiomyoma and accelerate abnormal uterine bleeding, GnRH agonists/antagonists, progestins, selective progesterone receptor modulators (SPRM), and aromatase inhibitors are available to inhibit estrogen actions and productions for leiomyoma-related symptoms [171,172]. LNG-IUD is the most common progestin treatment to induce amenorrhea and stop menorrhagia with anemia in up to 60% of premenopausal leiomyoma patients with minor side effects [177]. However, the efficacy of LNG-IUD significantly reduces in the patients with larger tumors (>3 cm) [177], which is similar to adenomyosis. Contraceptive pills combined with estrogen and progestin are commonly used for leiomyoma patients as well [171,172,178]. However, the efficacy of contraceptives for abnormal uterine and heavy bleeding is lower than that of LNG-IUD [177]. GnRH agonists are also effective in inducing amenorrhea and/or decreasing the size of leiomyoma [179,180]. However, the menopausal side effects induced by GnRH agonists are always a concern, therefore add-back hormonal therapies with GnRH agonists have been used for premenopausal women.

On the other hand, the effects of progesterone on leiomyoma can be different from other endometrial diseases. The reason is that progesterone/progestins stimulate cellular proliferation and the accumulation of ECM, which promote further development of uterine leiomyoma [8,168,181,182]. Clinical evidence also supports the mitogenic functions of progestins in leiomyoma patients. For example, the proliferation of leiomyoma tumors is most active during the secretory phase where progesterone secretion is high [183,184]. The combinations of estrogen and progestin therapies for menopausal women considerably enhance the growth of leiomyoma tumors compared with estrogen treatment alone [184]. A high dose of medroxyprogesterone acetate (MPA), which is a hormonal medication of the progestin, unfortunately increases leiomyoma growth [185]. Furthermore, an add-back therapy of progestins with GnRH agonists reverses the efficacy of GnRH agonists [186,187]. A study employing a xenograft model by grafting human leiomyoma tissues to the kidney capsule [181] shows that the size of xenografts of human leiomyoma tissues increases cell proliferation and volumes of ECM in response to 17β-estradiol plus progesterone, but this is not induced by the xenografts in which normal myometrium is implanted. Interestingly, 17β-estradiol or progesterone alone is unable to stimulate the growth of leiomyoma. The authors further demonstrate that while 17β-estradiol alone does not stimulate proliferation, 17β-estradiol induces PGR expression and supports progesterone action on the leiomyoma xenografts. The results suggest that estrogen and progesterone can directly stimulate leiomyoma cell proliferation based on the expression of PGR and ESR1 in the proliferating cells [8,181]. Estrogen and progesterone have also been reported to enhance ECM proteins such as collagen types I and II [188,189], and also stimulate ECM accumulation to increase the size of leiomyoma [181]. Thus, progesterone with a permissive role of estrogen is critical for cell proliferation and ECM accumulation to increase the size of leiomyoma tumors [8,168].

Additional studies of ECM in leiomyoma regulated by growth factors [190,191], cytokines [192], and steroid hormones [181,182] have been reported. A number of in vitro studies support that progestins stimulate cellular mechanisms to increase the production of growth factors summarized in [168]. Qiang et al. [182] have reported that ECM accumulation in leiomyoma is regulated by steroid hormones via the downregulation of miR-29b. The authors demonstrate that the expression of miR-29b is lower in leiomyoma compared with myometrium. Restoring miR-29b inhibits ECM accumulation to develop solid tumors. Although increased collagen expression by miR-29b is not sufficient for the transformation from myometrial cells to leiomyoma cells, 17β-estradiol and progesterone decrease miR-29b and increase multiple collagen expressions. These results suggest that miR-29R is one of the critical factors to produce ECM accumulation regulated by steroid hormones in leiomyoma. 

### 5.3. Genetic Alterations and Steroid Hormones in Uterine Leiomyoma

Compared with other endometrial diseases, leiomyomas show a high incidence of somatic mutations. Makinen’s group [193] has identified recurrent somatic mutations in *MED12* that drive leiomyoma development. Strikingly, approximately 70% of leiomyoma patients possess *MED12* mutations in leiomyoma tumors [193]. This finding has been further validated in multiple studies confirming the presence of *MED12* mutations, but depending on the study, the incidence varies between 48 and 92% [194]. HMGA2 overexpression is the second major genetic alteration accounting for approximately 10% in uterine leiomyoma cases [194,195,196], but its expression is limited in the leiomyoma without underlying *MED12* mutations [194,195,197,198,199]. *FH*-deficient or *COL4A5*/6 deletions have also been identified as unique genetic alterations without co-occurring with other alternations [198,200]. While *FH* and *COL4A5*/6 mutations have been characterized, the majority of uterine leiomyoma (80–90%) harbor *MED12* or *HMGA2* alterations [194]. Kurita’s group [201] has recently reported that subtypes of leiomyoma with either *MED12* mutations or HMGA2 overexpression required progesterone and 17β-estradiol to stimulate tumor growth. Another important finding is that leiomyomas with HMGA2 overexpression mainly consist of smooth muscle cells. Tumors with *MED12* mutations contain almost equal populations of smooth muscle cells and tumor-associated fibroblasts, although casual *MED12* mutations are present only in the smooth muscle cells [201]. Thus, the growth of smooth muscle cells is important in HMGA2 overexpressing leiomyoma. On the other hand, paracrine interactions between smooth muscle cells and tumor-associated fibroblasts are crucial for the progression of the *MED12* mutant leiomyoma, as tumor-associated fibroblasts do not carry *MED12* mutations. Interestingly, the growth of smooth muscle cells is driven by progesterone in both *MED12* mutant and HMGA2 overexpressing subtypes. In contrast, tumor-associated fibroblasts are stimulated by 17β-estradiol but not progesterone. Therefore, 17β-estradiol is likely to stimulate *MED12*-mutant smooth muscle cells to secrete paracrine factors that promote the growth of tumor-associated fibroblasts [201]. This study suggests that it is critical to consider the specific genetic alterations of leiomyoma subtypes when designing non-surgical therapeutic strategies and follow the tumor progression with different cell types due to the differential effects of steroid hormones on leiomyoma subtypes [201]. This group has also demonstrated that progesterone and 17β-estradiol activate MAPK and PI3K pathways with upregulation of IGF1 and IGF2 in the *MED12*-mutated leiomyoma [202]. 

RANKL has been identified as a progesterone responsive gene that is involved in the growth and progression of hormone-mediated leiomyoma [203,204]. Liu et al. [204] have shown that *RANKL* transcription is enhanced due to the hypomethylation of the regulatory element of PGR in leiomyoma stem cells, whereas higher DNA methylation at the PGR response element blocks PGR binding in the normal myometrium; leading to a decrease in RANKL expression. Furthermore, *MED12* mutation, especially at G44D, further stabilizes PGR binding at the regulator element of RANKL, indicating that a complex network constituted by DNA methylation and *MED12* mutations regulates the progesterone-mediated RANKL gene expression contributing to leiomyoma tumor development [204]. This group has further reported that the PGR gene locus and its genome-wide cistrome are hypermethylated in leiomyoma stem cells, repressing the expression of genes for progesterone-mediated leiomyoma stem cell differentiation [205].

WNT signaling has also been reported to mediate cellular processes in leiomyoma pathophysiology [197,206,207,208]. Several WNT ligands and other mediators have been overexpressed in leiomyoma to activate the WNT/CTNNB1 pathway to enhance leiomyoma progression [206]. Ono et al. [207] have reported that estrogen and progesterone activate canonical WNT/CTNNB1 signaling to stimulate cellular proliferation in the leiomyoma side population of stem-like cells, but this is not seen in the myometrial cells. The group also shows that WNT4 is overexpressed in CD34^+^/CD49b^−^ leiomyoma cells and can stimulate leiomyoma cell proliferation via WNT/CTNNB1 signaling and AKT [209]. Additionally, *MED12* mutations have been implicated in the misregulation of WNT/CTNNB1 signaling, providing additional linage between two common mechanisms of leiomyoma development [206,210,211,212]. 

## 6. Polycystic Ovary Syndrome (PCOS)

### 6.1. Disease Features and Current Treatments in PCOS

PCOS is known as a complex endocrine disorder characterized by ovulatory dysfunction, polycystic-appearing ovary, oligomenorrhea, and hyperandrogenism. PCOS is one of the common causes of female infertility occurring in 6–12% of reproductive-aged women, representing as many as 5 million women in the U.S. [213,214,215]. Approximately 75% of women with PCOS experience anovulation-related infertility and >50% of them suffer miscarriages and recurrent pregnancy loss [216,217,218]. As additional clinical complications of this disease, women with PCOS further develop type 2 diabetes and insulin resistance as well as increased risk of both cardiovascular diseases and endometrial cancer [213,214,219,220,221]. Although numerous factors impact the pathophysiology of PCOS, clinical, animal, and genetic studies support the involvement of abnormal neuroendocrine factors [222]. Dysregulation of hypothalamic GnRH and pituitary luteinizing hormone (LH) secretions [223,224], especially the high-frequency pulsatile release of GnRH following hypersecretion of LH, contribute to hyperandrogenisms [225]. In these women, the levels of the 17β-estradiol hormone are relatively normal, but their window of action can be prolonged [226,227]. Hypersecretion of LH and insulin with excess androgen arrests antral follicle growth and suppresses the follicle-stimulating hormone (FSH), leading to maturation inhibition in the follicles [228]. 

Currently, no universal treatments or drugs are available for the treatment of PCOS [229]. As risk factors for PCOS include being overweight, obesity, and type II diabetes with the consequences of insulin resistance, healthy lifestyle interventions are recommended to improve individual symptoms. On the other hand, anovulation and/or oligomenorrhea, a part of menstrual dysfunction, require hormonal therapies to have proper hormonal regulation not only in the ovary but also the uterus, which is also critical for the patients who seek fertility and successful pregnancy [229]. Oral contraceptive pills (combinations of estrogen and progesterone) and cyclic or continuous progesterone/progestin administration are often efficacious for patients with mild to moderate menstrual dysfunction [230]. On the other hand, it is known that chronic anovulation and oligomenorrhea elicit in women with PCOS when the endometrium is subject to prolonged estrogen exposure that is unopposed to subsequent progesterone signaling due to insufficient hormone production or PGR activation. This state results in endometrial hyperplasia, which can lead to endometrial cancer [221]. Indeed, PCOS patients have a significantly higher risk of developing endometrial cancer [221,231,232,233]. For those reasons, progestin/progesterone (i.e., MPA, norethindrone acetate, and micronized progesterone) have also been used to manage prolonged estrogen actions preventing endometrial hyperplasia [221,234]. However, the endometrium of women with PCOS often exhibits progesterone resistance, altering progesterone-regulated genes in the endometrium [21]. In fact, approximately 30% of PCOS patients fail to respond to progesterone-based therapy [235]. Therefore, it is important to understand endometrial pathophysiology in PCOS patients to improve poor reproductive outcomes and to prevent endometrial hyperplasia and/or cancer. 

### 6.2. Endometrial Progesterone Resistance in PCOS

Giudice and Lessey’s groups [21] have performed microarray analysis to determine differential gene expressions in the mid-secretory phase of endometrium from (1) PCOS patients treated with clomiphene citrate, an estrogen modulator, (2) PCOS patients treated with progesterone, and (3) normal patients. Their results have indicated that progesterone-regulated genes, such as *MIG6*, *LIF*, *GAB1*, *S100P,* and *CLDN4*, are downregulated in the endometrium from PCOS patients [21]. Specifically, MIG6 and GAB1, related to the EGF signaling pathway that is important for implantation and decidualization, are significantly reduced in the mid-secretory phase of PCOS patients despite the presence of progesterone. On the other hand, the estrogen signaling pathway associated with cell proliferation is aberrant [21], supporting a higher risk of endometrial hyperplasia and cancer [221]. The study summarizes the findings with minimal or absent progesterone responsiveness or progesterone resistance and elevated estrogen activity in the PCOS endometrium [21]. Furthermore, stromal cells isolated from PCOS endometrium have an aberrant decidualization response associated with increased expression and secretion of pro-inflammatory cytokines, chemokines, and MMPs [236]. Additionally, altered gene expression profiles in epithelial cells, stromal fibroblasts, and mesenchymal stem cells have revealed that inflammatory and pro-oncogenic changes negatively impact endometrial functions in women with PCOS [22]. 

The absence of progesterone responsiveness and/or progesterone resistance could be explained by altered PGR expression. Quezada et al. [237] have reported that PGR is higher in the epithelium during the mid-secretory phase in PCOS patients. PRA expression is elevated in the proliferative endometrium in PCOS patients [238]. The altered PRA/PRB ratio has also been observed in the proliferative endometrium with PCOS [237,238]. However, it is still unclear whether direct correlations exist between loss of progesterone responsiveness and the altered expression of PGR and specific isoforms in the PCOS endometrium.

### 6.3. Metformin and Progesterone Resistance in PCOS

Metformin is an insulin sensitizer that has been widely used not only for type 2 diabetes but PCOS [239,240]. Treatment of PCOS patients with metformin improves menstrual irregularity and anovulation [239,240], resulting in inhibiting cytokine production, CYP19A1 (aromatase) activity, and endometrial cell proliferation [241]. Metformin can enhance PGR expression via inhibition of overactivated mTOR signaling [242]. Furthermore, the combination of metformin and oral contraceptives is effective in reducing progestin-resistant endometrial hyperplasia [243,244]. A few experimental studies and one clinical study support the use of metformin in endometriosis [245]. Further investigation is necessary to understand the molecular mechanisms to improve endometrial dysfunctions (endometrial hyperplasia and progesterone resistance) with insulin resistance and can provide the undiscovered impeccable pathophysiology of PCOS as well as other endometrial diseases. 

## 7. Endometrial Hyperplasia with or without Atypia

### 7.1. Characterization of Endometrial Hyperplasia

Endometrial hyperplasia is the condition of excessive proliferation of epithelial cells and thickening of the endometrium, usually resulting from chronic unopposed estrogen exposure associated with deficiency of progesterone [246,247,248]. Hyperplastic endometrium is induced by hormone imbalance, however, it might further develop into atypical hyperplasia (AH)/endometrioid intraepithelial neoplasia (EIN) [249]. AH has been histologically defined by complex hyperplastic glands composed of enlarged and irregular-shaped nuclei, displaying stratification and loss of polarity, and resembling the morphological feature in endometrial cancer [250,251]. Endometrial hyperplasia with or without cytologic atypia has been classified in the 2014 World Health Organization (WHO) classification for the purpose of clinical practice and the choice of treatment [250]. AH raises a significant risk of developing endometrial cancer [252,253,254]. Kurman et al. [255] has reported that 1.6% of patients with endometrial hyperplasia progress to carcinomas compared with 23% of patients with AH [255]. The study from Lacey et al. shows that AH/EIN has a nearly 40% probability of developing endometrial cancer [256]. Several case studies also report 14% up to 43% concurrent associations with endometrial carcinomas and AH [257,258,259]. These studies support that AH/EIN are considered precursor or premalignant tumors specifically for endometrioid endometrial carcinomas (EEC) [250,254,260]. EEC accounts for >80% of all endometrial carcinomas and usually develop in premenopausal and peri-menopausal women, whereas serous uterine carcinomas account for <10% and are highly aggressive, estrogen-independent, and diagnosed mainly in postmenopausal women [249,261,262,263,264,265]. EEC are well known to be correlated with genetic alterations in *PTEN*, *KRAS*, *CTNNB1, ARID1A,* and *PIK3CA* [120,266]. Although multiple combinations of mutations have been observed in EEC, approximately 65% of EEC harbor *PTEN* mutations [266], which are the most frequent somatic mutation in EEC [249,267,268]. Somatic *PTEN* mutations have been observed not only in EEC but also in hyperplastic glands and AH/EIN [249,269]. A recent genome-wide mutation analysis from Li et al. [251] has reported that the common mutations between AH and EEC vary from 0.1% to 82%. Microsatellite stable AH have fewer cancer-driving mutations than EEC, and 79% of microsatellite stable EEC gain cancer driver mutations related to *PTEN*, *CTNNB1, ARID1A*, *CHD4,* and *PIK3CA*, indicating that some AH lesions are immediate precursors of EEC, and their progression depends on the acquisition of additional cancer driver mutations. In the genetically engineered mouse models of endometrial cancer, conditional ablation of *PTEN* in epithelial cells and/or endometrial cells induces AH/EIN and/or carcinomas [8,270,271,272]. Thus, *PTEN* mutations in the endometrium can be a major driver of AH and appear to initiate the precursor of EEC, whereas a single mutation of *Pik3ca^E545K^* [270] or *Arid1a* [273,274] is insufficient to induce endometrial hyperplasia, AH/EIN, or cancerous lesions. On the other hand, *PTEN* mutations alone are considered to be insufficient to initiate malignant tumorigenesis unless other molecular alterations are acquired [249]. Genetically engineered mouse models have shown that the combinations with *Pten* ablation with mutations of *Pik3ca* [270], *Arid1a* [273], or *Kras^G12D^* [272] promote and aggressively develop invasive endometrial carcinomas, whereas solely *PTEN* loss does not or takes a long time to induce malignant tumors. Thus, *PTEN* ablation is likely an early event to induce endometrial hyperplasia and/or AH/EIN, but *PTEN* mutations can further accelerate the progression to endometrial carcinoma with other mutations. Interestingly, *PTEN* mutations with estrogen exposure result in an increased incidence of endometrial carcinomas [275]. However, *Pten^+/−^* mice with oophorectomies often develop hyperplastic lesions, and *Pten^+/−^ Esr*^−/−^ mice also exhibit atypical hyperplasia and endometrial tumorigenesis, indicating that AH/EIN induced by *PTEN* mutations is independent of estrogen and estrogen signaling [275]. 

### 7.2. Hormonal Therapy and Clinical Perspectives for Endometrial Hyperplasia, AH/EIN, and Endometrial Cancer

Because most of the patients with endometrial hyperplasia, AH/EIN, and/or carcinomas present with abnormal uterine bleeding, endometrial cancer is generally diagnosed at the early stages [248,254]. However, abnormal bleeding can be induced by many different causes. After ruling out other sources and causes of abnormal bleeding by physical and histological examinations and tests, removing the uterus (hysterectomy) and ovaries and fallopian tubes (bilateral salpingo-oophorectomy) is a primary option and the current standard for the treatment of endometrial cancer [248,254]. Of course, cytoreductive surgery, including hysterectomy and oophorectomy, is not the option for patients who wish to preserve fertility. It has been reported that hormonal therapy, mainly progestins, shows successful efficacy in resolving premalignant endometrial hyperplasia and/or early stages of EEC [276]. Long-term treatment with MPA and megestrol acerate resolve approximately 75% of endometrial hyperplasia [277,278,279,280,281]. LNG-IUD is effective in regressing endometrial hyperplasia after 2 years of LNG-IUD insertion [282]. Favorable responses to endometrial cancer depend on ESR and PGR expression, low-grade EEC, and low disease burden [263,283]. Hormonal therapy is often considered for women with AH. However, the efficacy of progestin therapy is limited for many AH patients [248,254]. The variability in response to progestins is due to the heterogeneity of AH lesions with different genetic mutation statuses as described above. If gene mutation(s) initiates AH and/or endometrial hyperplasia without dysregulation of steroid hormones, hormonal therapy is less likely to be effective. It will be crucial to understand the pathophysiology of endometrial hyperplasia and AH/EIC, as well as the cause and mechanism of transformation from endometrial hyperplasia to AH/EIC. Nevertheless, untreated premalignant lesions of the endometrium are highly likely to progress to endometrial cancer [254]. As it is estimated that there is an interval range of 4–7 years from the diagnosis from AH to EEC [256,284], preventing the progression from AH to EEC by hormonal therapy (progestin treatment) might be an option for patients who wish to preserve fertility using indicators of ESR1 and PGR expression.

**Table 1:** Gynecological diseases.

Gynecological Diseases	Diseases Features	Progesterone Actions/PGR Signaling	Mutations	Major Symptoms	Common Treatment Options
Endometriosis	Endometrial-like tissues outside of the uterus Hemosiderin Extensive adhesion	Dysregulated (Decreased) [16,17,70,71,88,89,90,91,92,93,94,95,96,97,98,99,105,106,109,110,111,112,114,115,116,117,119,124,125,126]	*KRAS* *PIK3CA* *ARID1A* *PPP2R1A* [30,31,32,34]	Dysmenorrhea Chronic pelvic pain Dyspareunia Heavy bleeding Infertility	GnRH agonists and antagonists Combined oral contraceptives Non-steroidal anti-inflammatory drugs Progestins (LNG-IUD, implant, injection, pills, etc.) Surgical removal and destruction (laparoscopy) Hysterectomy and/or oophorectomy [44,45,46,47,48,49,50,51,52,53,54,55]
Adenomyosis	Endometrial-like tissues in	Dysregulated (Decreased)	*KRAS*	Menorrhagia with heavy bleeding Non-steroidal anti-inflammatory drugs	
	myometrium	[136,163]	*PIK3CA*	Chronic pelvic pain	GnRH agonists and antagonists
			[31]	Implantation failures	Progestins (LNG-IUD, implant, injection, pills, etc.)
			Miscarriages		Hysterectomy Androgenic hormones [132,151,152,153,154,155,156,157,158,159,160,161,162]
Leiomyoma	Benign tumors with smooth muscle cells and fibroblasts	Progesterone and 17β-estradiol act as stimulators for tumor growth [181,182,184,185,201,202,203,204,205,206,207]	*MED12* HMGA2 overexpression [193,194,195,196,197,198,199]	Menorrhagia with heavy bleeding Pelvic pain and pressure Constipation Frequent urination Infertility	GnRH agonists and antagonists Progestins (LNG-IUD, implant, injection, pills, etc.) Uterine artery embolization Myomectomy Hysterectomy [171,172,174,175,176,177,178,179,180]
PCOS	Endocrine disorder with ovulatory dysfunction and polycystic ovary Oligomenorrhea Hyperandrogenism	Dysregulated (Decreased) [21,22,237,238]		Infertility Miscarriages Develop type 2 diabetes with insulin resistance	Combined oral contraceptives Progestins Metformin Aromatase inhibitor [229,230,234,235,239,240,243,244]
Endometrial Hyperplasia	Excessive proliferation of epithelial cells and thickening of the endometrium	Dysregulated (Decreased) [246,247,248]	*PTEN* *KRAS* *PIK3CA* *ARID1A* [249,251,269]	Abnormal menstruation Heavy bleeding	Progestins (LNG-IUD, implant, injection, pills, etc.) Combined oral contraceptives [248,254,276,277,278,279,280,281,282]

## 8. Conclusions

While each gynecological disease described above may have different origins, mechanisms, and etiology, there are some similarities, chiefly dysregulated steroid hormone signaling. This may stem from a disruption of hormone production, progesterone resistance, altered hormone-dependent gene expression, common somatic gene mutations, and/or side effects of current hormonal treatments and approaches for one disease that establish or worsen another. Sadly, most of these patients have been battling disease-associated chronic pelvic pain, dysmenorrhea, menorrhagia, and/or fertility issues, reducing their quality of life, with few perfect therapy strategies to abrogate their disorders. The dynamic changes of endometrial tissues in the menstrual cycle regulated by responses of steroid hormones are complex mechanisms. Because menstrual cycles repeatably occur and alter the local endometrial environment, the local hormonal environment cannot be precisely defined in each cycle. Endometrial proliferation, differentiation, shedding, and regeneration are associated with massive inflammation which further affects endometrial hormonal responsiveness, including epigenomic and transcriptomic interplay. While numerous studies reveal the mechanisms of hormone unresponsiveness and progesterone resistance, we still do not know the initiation and establishment of each gynecological disorder and how they can be therapeutically treated. The studies from Fazleabas’ group have indicated that the development of progesterone resistance is a gradual process and becomes evident at least 6 months after disease induction in the baboon model of endometriosis [285]. Thus, the establishment of disease (endometriotic lesions) can further affect the eutopic endometrial environment. Recent studies also indicate that somatic gene mutations can be drivers to promote disease establishment; leiomyoma and uterine hyperplasia with AH/EIC are especially likely initiated by gene mutations. Somatic mutations have been observed in the endometrium with endometriosis and adenomyosis and are also related to potentially transforming into malignant tumors. In this context, it would be important to understand the further mechanisms of disease pathophysiology and develop personalized medicine depending on the alterations of complex mechanisms, symptoms, and future goals for patients who desire fertility. 

## Data Availability

Not applicable.

## References

[1] Graham J.D., Clarke C.L. (1997). Physiological action of progesterone in target tissues. Endocr. Rev..

[2] Lydon J.P., DeMayo F.J., Funk C.R., Mani S.K., Hughes A.R., Montgomery C.A., Shyamala G., Conneely O.M., O’Malley B.W. (1995). Mice lacking progesterone receptor exhibit pleiotropic reproductive abnormalities. Genes Dev..

[3] Patel B., Elguero S., Thakore S., Dahoud W., Bedaiwy M., Mesiano S. (2015). Role of nuclear progesterone receptor isoforms in uterine pathophysiology. Hum. Reprod. Update.

[4] Kastner P., Krust A., Turcotte B., Stropp U., Tora L., Gronemeyer H., Chambon P. (1990). Two distinct estrogen-regulated promoters generate transcripts encoding the two functionally different human progesterone receptor forms A and B. EMBO J..

[5] Mulac-Jericevic B., Mullinax R.A., DeMayo F.J., Lydon J.P., Conneely O.M. (2000). Subgroup of reproductive functions of progesterone mediated by progesterone receptor-B isoform. Science.

[6] Mulac-Jericevic B., Lydon J.P., DeMayo F.J., Conneely O.M. (2003). Defective mammary gland morphogenesis in mice lacking the progesterone receptor B isoform. Proc. Natl. Acad. Sci. USA.

[7] DeMayo F.J., Zhao B., Takamoto N., Tsai S.Y. (2002). Mechanisms of action of estrogen and progesterone. Ann. N. Y. Acad. Sci..

[8] Kim J.J., Kurita T., Bulun S.E. (2013). Progesterone action in endometrial cancer, endometriosis, uterine fibroids, and breast cancer. Endocr. Rev..

[9] Marquardt R.M., Kim T.H., Shin J.H., Jeong J.W. (2019). Progesterone and Estrogen Signaling in the Endometrium: What Goes Wrong in Endometriosis?. Int. J. Mol. Sci..

[10] Gellersen B., Brosens I.A., Brosens J.J. (2007). Decidualization of the human endometrium: Mechanisms, functions, and clinical perspectives. Semin. Reprod. Med..

[11] Gellersen B., Brosens J.J. (2014). Cyclic decidualization of the human endometrium in reproductive health and failure. Endocr. Rev..

[12] Al-Sabbagh M., Lam E.W., Brosens J.J. (2012). Mechanisms of endometrial progesterone resistance. Mol. Cell Endocrinol..

[13] McKinnon B., Mueller M., Montgomery G. (2018). Progesterone Resistance in Endometriosis: An Acquired Property?. Trends Endocrinol. Metab..

[14] Patel B.G., Rudnicki M., Yu J., Shu Y., Taylor R.N. (2017). Progesterone resistance in endometriosis: Origins, consequences and interventions. Acta Obstet. Gynecol. Scand..

[15] Li X., Feng Y., Lin J.F., Billig H., Shao R. (2014). Endometrial progesterone resistance and PCOS. J. Biomed. Sci..

[16] Burney R.O., Talbi S., Hamilton A.E., Vo K.C., Nyegaard M., Nezhat C.R., Lessey B.A., Giudice L.C. (2007). Gene expression analysis of endometrium reveals progesterone resistance and candidate susceptibility genes in women with endometriosis. Endocrinology.

[17] Kao L.C., Germeyer A., Tulac S., Lobo S., Yang J.P., Taylor R.N., Osteen K., Lessey B.A., Giudice L.C. (2003). Expression profiling of endometrium from women with endometriosis reveals candidate genes for disease-based implantation failure and infertility. Endocrinology.

[18] Houshdaran S., Nezhat C.R., Vo K.C., Zelenko Z., Irwin J.C., Giudice L.C. (2016). Aberrant Endometrial DNA Methylome and Associated Gene Expression in Women with Endometriosis. Biol. Reprod..

[19] Houshdaran S., Oke A.B., Fung J.C., Vo K.C., Nezhat C., Giudice L.C. (2020). Steroid hormones regulate genome-wide epigenetic programming and gene transcription in human endometrial cells with marked aberrancies in endometriosis. PLoS Genet..

[20] Moustafa S., Young S.L. (2020). Diagnostic and therapeutic options in recurrent implantation failure. F1000Research.

[21] Savaris R.F., Groll J.M., Young S.L., DeMayo F.J., Jeong J.W., Hamilton A.E., Giudice L.C., Lessey B.A. (2011). Progesterone resistance in PCOS endometrium: A microarray analysis in clomiphene citrate-treated and artificial menstrual cycles. J. Clin. Endocrinol. Metab..

[22] Piltonen T.T., Chen J., Erikson D.W., Spitzer T.L., Barragan F., Rabban J.T., Huddleston H., Irwin J.C., Giudice L.C. (2013). Mesenchymal stem/progenitors and other endometrial cell types from women with polycystic ovary syndrome (PCOS) display inflammatory and oncogenic potential. J. Clin. Endocrinol. Metab..

[23] Tamaresis J.S., Irwin J.C., Goldfien G.A., Rabban J.T., Burney R.O., Nezhat C., DePaolo L.V., Giudice L.C. (2014). Molecular classification of endometriosis and disease stage using high-dimensional genomic data. Endocrinology.

[24] Guo S.W. (2009). Epigenetics of endometriosis. Mol. Hum. Reprod..

[25] Meyer J.L., Zimbardi D., Podgaec S., Amorim R.L., Abrao M.S., Rainho C.A. (2014). DNA methylation patterns of steroid receptor genes ESR1, ESR2 and PGR in deep endometriosis compromising the rectum. Int. J. Mol. Med..

[26] Rocha-Junior C.V., Da Broi M.G., Miranda-Furtado C.L., Navarro P.A., Ferriani R.A., Meola J. (2019). Progesterone Receptor B (PGR-B) Is Partially Methylated in Eutopic Endometrium From Infertile Women With Endometriosis. Reprod. Sci..

[27] Wu Y., Strawn E., Basir Z., Halverson G., Guo S.W. (2006). Promoter hypermethylation of progesterone receptor isoform B (PR-B) in endometriosis. Epigenetics.

[28] Xue Q., Lin Z., Yin P., Milad M.P., Cheng Y.H., Confino E., Reierstad S., Bulun S.E. (2007). Transcriptional activation of steroidogenic factor-1 by hypomethylation of the 5′ CpG island in endometriosis. J. Clin. Endocrinol. Metab..

[29] Wu Y., Halverson G., Basir Z., Strawn E., Yan P., Guo S.W. (2005). Aberrant methylation at HOXA10 may be responsible for its aberrant expression in the endometrium of patients with endometriosis. Am. J. Obstet. Gynecol..

[30] Anglesio M.S., Papadopoulos N., Ayhan A., Nazeran T.M., Noe M., Horlings H.M., Lum A., Jones S., Senz J., Seckin T. (2017). Cancer-Associated Mutations in Endometriosis without Cancer. N. Engl. J. Med..

[31] Inoue S., Hirota Y., Ueno T., Fukui Y., Yoshida E., Hayashi T., Kojima S., Takeyama R., Hashimoto T., Kiyono T. (2019). Uterine adenomyosis is an oligoclonal disorder associated with KRAS mutations. Nat. Commun..

[32] Li X., Zhang Y., Zhao L., Wang L., Wu Z., Mei Q., Nie J., Li X., Li Y., Fu X. (2014). Whole-exome sequencing of endometriosis identifies frequent alterations in genes involved in cell adhesion and chromatin-remodeling complexes. Hum. Mol. Genet..

[33] Moore L., Leongamornlert D., Coorens T.H.H., Sanders M.A., Ellis P., Dentro S.C., Dawson K.J., Butler T., Rahbari R., Mitchell T.J. (2020). The mutational landscape of normal human endometrial epithelium. Nature.

[34] Suda K., Nakaoka H., Yoshihara K., Ishiguro T., Tamura R., Mori Y., Yamawaki K., Adachi S., Takahashi T., Kase H. (2018). Clonal Expansion and Diversification of Cancer-Associated Mutations in Endometriosis and Normal Endometrium. Cell Rep..

[35] Bulun S.E., Yilmaz B.D., Sison C., Miyazaki K., Bernardi L., Liu S., Kohlmeier A., Yin P., Milad M., Wei J. (2019). Endometriosis. Endocr. Rev..

[36] Taylor H.S., Kotlyar A.M., Flores V.A. (2021). Endometriosis is a chronic systemic disease: Clinical challenges and novel innovations. Lancet.

[37] Zondervan K.T., Becker C.M., Missmer S.A. (2020). Endometriosis. N. Engl. J. Med..

[38] Zondervan K.T., Becker C.M., Koga K., Missmer S.A., Taylor R.N., Vigano P. (2018). Endometriosis. Nat. Rev. Dis. Primers.

[39] Sampson J.A. (1927). Metastatic or Embolic Endometriosis, due to the Menstrual Dissemination of Endometrial Tissue into the Venous Circulation. Am. J. Pathol..

[40] Halme J., Hammond M.G., Hulka J.F., Raj S.G., Talbert L.M. (1984). Retrograde menstruation in healthy women and in patients with endometriosis. Obstet. Gynecol..

[41] Shafrir A.L., Farland L.V., Shah D.K., Harris H.R., Kvaskoff M., Zondervan K., Missmer S.A. (2018). Risk for and consequences of endometriosis: A critical epidemiologic review. Best Pract. Res. Clin. Obstet. Gynaecol..

[42] Nnoaham K.E., Hummelshoj L., Webster P., d’Hooghe T., de Cicco Nardone F., de Cicco Nardone C., Jenkinson C., Kennedy S.H., Zondervan K.T., World Endometriosis Research Foundation Global Study of Women’s Health Consortium (2011). Impact of endometriosis on quality of life and work productivity: A multicenter study across ten countries. Fertil. Steril..

[43] Simoens S., Dunselman G., Dirksen C., Hummelshoj L., Bokor A., Brandes I., Brodszky V., Canis M., Colombo G.L., DeLeire T. (2012). The burden of endometriosis: Costs and quality of life of women with endometriosis and treated in referral centres. Hum. Reprod..

[44] Vercellini P., Vigano P., Somigliana E., Fedele L. (2014). Endometriosis: Pathogenesis and treatment. Nat. Rev. Endocrinol..

[45] Dunselman G.A., Vermeulen N., Becker C., Calhaz-Jorge C., D’Hooghe T., De Bie B., Heikinheimo O., Horne A.W., Kiesel L., Nap A. (2014). ESHRE guideline: Management of women with endometriosis. Hum. Reprod..

[46] Vercellini P., Bracco B., Mosconi P., Roberto A., Alberico D., Dhouha D., Somigliana E. (2016). Norethindrone acetate or dienogest for the treatment of symptomatic endometriosis: A before and after study. Fertil. Steril..

[47] Agarwal S.K., Chapron C., Giudice L.C., Laufer M.R., Leyland N., Missmer S.A., Singh S.S., Taylor H.S. (2019). Clinical diagnosis of endometriosis: A call to action. Am. J. Obstet. Gynecol..

[48] Abou-Setta A.M., Houston B., Al-Inany H.G., Farquhar C. (2013). Levonorgestrel-releasing intrauterine device (LNG-IUD) for symptomatic endometriosis following surgery. Cochrane Database Syst. Rev..

[49] Barra F., Grandi G., Tantari M., Scala C., Facchinetti F., Ferrero S. (2019). A comprehensive review of hormonal and biological therapies for endometriosis: Latest developments. Expert Opin. Biol. Ther..

[50] Giudice L.C. (2010). Clinical practice. Endometriosis. N. Engl. J. Med..

[51] As-Sanie S., Black R., Giudice L.C., Gray Valbrun T., Gupta J., Jones B., Laufer M.R., Milspaw A.T., Missmer S.A., Norman A. (2019). Assessing research gaps and unmet needs in endometriosis. Am. J. Obstet. Gynecol..

[52] DeCherney A.H. (1992). Endometriosis: Recurrence and retreatment. Clin. Ther..

[53] Evers J.L., Dunselman G.A., Land J.A., Bouckaert P.X. (1991). Is there a solution for recurrent endometriosis?. Br. J. Clin. Pract. Suppl..

[54] Practice Committee of American Society for Reproductive Medicine (2008). Treatment of pelvic pain associated with endometriosis. Fertil. Steril..

[55] Waller K.G., Shaw R.W. (1993). Gonadotropin-releasing hormone analogues for the treatment of endometriosis: Long-term follow-up. Fertil. Steril..

[56] Reis F.M., Coutinho L.M., Vannuccini S., Batteux F., Chapron C., Petraglia F. (2020). Progesterone receptor ligands for the treatment of endometriosis: The mechanisms behind therapeutic success and failure. Hum. Reprod. Update.

[57] Attia G.R., Zeitoun K., Edwards D., Johns A., Carr B.R., Bulun S.E. (2000). Progesterone receptor isoform A but not B is expressed in endometriosis. J. Clin. Endocrinol. Metab..

[58] Bedaiwy M.A., Dahoud W., Skomorovska-Prokvolit Y., Yi L., Liu J.H., Falcone T., Hurd W.W., Mesiano S. (2015). Abundance and Localization of Progesterone Receptor Isoforms in Endometrium in Women with and without Endometriosis and in Peritoneal and Ovarian Endometriotic Implants. Reprod. Sci..

[59] Wu Y., Starzinski-Powitz A., Guo S.W. (2008). Prolonged stimulation with tumor necrosis factor-alpha induced partial methylation at PR-B promoter in immortalized epithelial-like endometriotic cells. Fertil. Steril..

[60] Gellersen B., Fernandes M.S., Brosens J.J. (2009). Non-genomic progesterone actions in female reproduction. Hum. Reprod. Update.

[61] Gellersen B., Brosens J. (2003). Cyclic AMP and progesterone receptor cross-talk in human endometrium: A decidualizing affair. J. Endocrinol..

[62] Jones M.C., Fusi L., Higham J.H., Abdel-Hafiz H., Horwitz K.B., Lam E.W., Brosens J.J. (2006). Regulation of the SUMO pathway sensitizes differentiating human endometrial stromal cells to progesterone. Proc. Natl. Acad. Sci. USA.

[63] Prentice A., Randall B.J., Weddell A., McGill A., Henry L., Horne C.H., Thomas E.J. (1992). Ovarian steroid receptor expression in endometriosis and in two potential parent epithelia: Endometrium and peritoneal mesothelium. Hum. Reprod..

[64] Broi M.G.D., Rocha C.V.J., Meola J., Martins W.P., Carvalho F.M., Ferriani R.A., Navarro P.A. (2017). Expression of PGR, HBEGF, ITGAV, ITGB3 and SPP1 genes in eutopic endometrium of infertile women with endometriosis during the implantation window: A pilot study. JBRA Assist. Reprod..

[65] Zanatta A., Pereira R.M., Rocha A.M., Cogliati B., Baracat E.C., Taylor H.S., Motta E.L., Serafini P.C. (2015). The relationship among HOXA10, estrogen receptor alpha, progesterone receptor, and progesterone receptor B proteins in rectosigmoid endometriosis: A tissue microarray study. Reprod. Sci..

[66] Santamaria X., Mas A., Cervello I., Taylor H., Simon C. (2018). Uterine stem cells: From basic research to advanced cell therapies. Hum. Reprod. Update.

[67] Symons L.K., Miller J.E., Kay V.R., Marks R.M., Liblik K., Koti M., Tayade C. (2018). The Immunopathophysiology of Endometriosis. Trends Mol. Med..

[68] Han S.J., Jung S.Y., Wu S.P., Hawkins S.M., Park M.J., Kyo S., Qin J., Lydon J.P., Tsai S.Y., Tsai M.J. (2015). Estrogen Receptor beta Modulates Apoptosis Complexes and the Inflammasome to Drive the Pathogenesis of Endometriosis. Cell.

[69] Lessey B.A., Kim J.J. (2017). Endometrial receptivity in the eutopic endometrium of women with endometriosis: It is affected, and let me show you why. Fertil. Steril..

[70] Aghajanova L., Horcajadas J.A., Weeks J.L., Esteban F.J., Nezhat C.N., Conti M., Giudice L.C. (2010). The protein kinase A pathway-regulated transcriptome of endometrial stromal fibroblasts reveals compromised differentiation and persistent proliferative potential in endometriosis. Endocrinology.

[71] Taylor H.S., Bagot C., Kardana A., Olive D., Arici A. (1999). HOX gene expression is altered in the endometrium of women with endometriosis. Hum. Reprod..

[72] Garcia-Alonso L., Handfield L.F., Roberts K., Nikolakopoulou K., Fernando R.C., Gardner L., Woodhams B., Arutyunyan A., Polanski K., Hoo R. (2021). Mapping the temporal and spatial dynamics of the human endometrium in vivo and in vitro. Nat. Genet..

[73] Tan Y., Flynn W., Sivajothi S., Luo S., Bozal S., Luciano A., Robson P., Luciano D., Courtois E. (2021). Single cell analysis of endometriosis reveals a coordinated transcriptional program driving immunotolerance and angiogenesis across eutopic and ectopic tissues. bioRxiv.

[74] Fonseca M., Wright K., Lin X., Abbasi F., Haro M., Sun J., Hernandez L., Orr N., Hong J., Choi-Kuaea Y. (2021). A cellular and molecular portrait of endometriosis subtypes. bioRxiv.

[75] Rowan B.G., O’Malley B.W. (2000). Progesterone receptor coactivators. Steroids.

[76] Han S.J., DeMayo F.J., O’Malley B.W. (2007). Dynamic regulation of progesterone receptor activity in female reproductive tissues. Progestins and the Mammary Gland.

[77] Xu J., Qiu Y., DeMayo F.J., Tsai S.Y., Tsai M.J., O’Malley B.W. (1998). Partial hormone resistance in mice with disruption of the steroid receptor coactivator-1 (SRC-1) gene. Science.

[78] Mukherjee A., Soyal S.M., Fernandez-Valdivia R., Gehin M., Chambon P., Demayo F.J., Lydon J.P., O’Malley B.W. (2006). Steroid receptor coactivator 2 is critical for progesterone-dependent uterine function and mammary morphogenesis in the mouse. Mol. Cell Biol..

[79] Xu J., Liao L., Ning G., Yoshida-Komiya H., Deng C., O’Malley B.W. (2000). The steroid receptor coactivator SRC-3 (p/CIP/RAC3/AIB1/ACTR/TRAM-1) is required for normal growth, puberty, female reproductive function, and mammary gland development. Proc. Natl. Acad. Sci. USA.

[80] Han S.J., DeMayo F.J., Xu J., Tsai S.Y., Tsai M.J., O’Malley B.W. (2006). Steroid receptor coactivator (SRC)-1 and SRC-3 differentially modulate tissue-specific activation functions of the progesterone receptor. Mol. Endocrinol..

[81] Jeong J.W., Lee K.Y., Han S.J., Aronow B.J., Lydon J.P., O’Malley B.W., DeMayo F.J. (2007). The p160 steroid receptor coactivator 2, SRC-2, regulates murine endometrial function and regulates progesterone-independent and -dependent gene expression. Endocrinology.

[82] Han S.J., Jeong J., Demayo F.J., Xu J., Tsai S.Y., Tsai M.J., O’Malley B.W. (2005). Dynamic cell type specificity of SRC-1 coactivator in modulating uterine progesterone receptor function in mice. Mol. Cell. Biol..

[83] Han S.J., Hawkins S.M., Begum K., Jung S.Y., Kovanci E., Qin J., Lydon J.P., DeMayo F.J., O’Malley B.W. (2012). A new isoform of steroid receptor coactivator-1 is crucial for pathogenic progression of endometriosis. Nat. Med..

[84] Takamoto N., Zhao B., Tsai S.Y., DeMayo F.J. (2002). Identification of Indian hedgehog as a progesterone-responsive gene in the murine uterus. Mol. Endocrinol..

[85] Matsumoto H., Zhao X., Das S.K., Hogan B.L., Dey S.K. (2002). Indian hedgehog as a progesterone-responsive factor mediating epithelial-mesenchymal interactions in the mouse uterus. Dev. Biol..

[86] Lee K., Jeong J., Kwak I., Yu C.T., Lanske B., Soegiarto D.W., Toftgard R., Tsai M.J., Tsai S., Lydon J.P. (2006). Indian hedgehog is a major mediator of progesterone signaling in the mouse uterus. Nat. Genet..

[87] Kurihara I., Lee D.K., Petit F.G., Jeong J., Lee K., Lydon J.P., DeMayo F.J., Tsai M.J., Tsai S.Y. (2007). COUP-TFII mediates progesterone regulation of uterine implantation by controlling ER activity. PLoS Genet..

[88] Smith K., Alnifaidy R., Wei Q., Nieman L.K. (2011). Endometrial Indian hedgehog expression is decreased in women with endometriosis. Fertil. Steril..

[89] Lin S.C., Li Y.H., Wu M.H., Chang Y.F., Lee D.K., Tsai S.Y., Tsai M.J., Tsai S.J. (2014). Suppression of COUP-TFII by proinflammatory cytokines contributes to the pathogenesis of endometriosis. J. Clin. Endocrinol. Metab..

[90] Liang Y., Li Y., Liu K., Chen P., Wang D. (2016). Expression and Significance of WNT4 in Ectopic and Eutopic Endometrium of Human Endometriosis. Reprod. Sci..

[91] Jeong J.W., Lee H.S., Lee K.Y., White L.D., Broaddus R.R., Zhang Y.W., Vande Woude G.F., Giudice L.C., Young S.L., Lessey B.A. (2009). Mig-6 modulates uterine steroid hormone responsiveness and exhibits altered expression in endometrial disease. Proc. Natl. Acad. Sci. USA.

[92] Yoo J.Y., Kim T.H., Lee J.H., Dunwoodie S.L., Ku B.J., Jeong J.W. (2015). Mig-6 regulates endometrial genes involved in cell cycle and progesterone signaling. Biochem. Biophys. Res. Commun..

[93] Su R.W., Strug M.R., Joshi N.R., Jeong J.W., Miele L., Lessey B.A., Young S.L., Fazleabas A.T. (2015). Decreased Notch pathway signaling in the endometrium of women with endometriosis impairs decidualization. J. Clin. Endocrinol. Metab..

[94] Yoo J.Y., Shin H., Kim T.H., Choi W.S., Ferguson S.D., Fazleabas A.T., Young S.L., Lessey B.A., Ha U.H., Jeong J.W. (2014). CRISPLD2 is a target of progesterone receptor and its expression is decreased in women with endometriosis. PLoS ONE.

[95] Franco H.L., Dai D., Lee K.Y., Rubel C.A., Roop D., Boerboom D., Jeong J.W., Lydon J.P., Bagchi I.C., Bagchi M.K. (2011). WNT4 is a key regulator of normal postnatal uterine development and progesterone signaling during embryo implantation and decidualization in the mouse. FASEB J..

[96] Benson G.V., Lim H., Paria B.C., Satokata I., Dey S.K., Maas R.L. (1996). Mechanisms of reduced fertility in Hoxa-10 mutant mice: Uterine homeosis and loss of maternal Hoxa-10 expression. Development.

[97] Lim H., Ma L., Ma W.G., Maas R.L., Dey S.K. (1999). Hoxa-10 regulates uterine stromal cell responsiveness to progesterone during implantation and decidualization in the mouse. Mol. Endocrinol..

[98] Brosens J.J., Gellersen B. (2006). Death or survival—Progesterone-dependent cell fate decisions in the human endometrial stroma. J. Mol. Endocrinol..

[99] Vasquez Y.M., Wang X., Wetendorf M., Franco H.L., Mo Q., Wang T., Lanz R.B., Young S.L., Lessey B.A., Spencer T.E. (2018). FOXO1 regulates uterine epithelial integrity and progesterone receptor expression critical for embryo implantation. PLoS Genet..

[100] Afshar Y., Jeong J.W., Roqueiro D., DeMayo F., Lydon J., Radtke F., Radnor R., Miele L., Fazleabas A. (2012). Notch1 mediates uterine stromal differentiation and is critical for complete decidualization in the mouse. FASEB J..

[101] Afshar Y., Miele L., Fazleabas A.T. (2012). Notch1 is regulated by chorionic gonadotropin and progesterone in endometrial stromal cells and modulates decidualization in primates. Endocrinology.

[102] Brown D.M., Lee H.C., Liu S., Quick C.M., Fernandes L.M., Simmen F.A., Tsai S.J., Simmen R.C.M. (2018). Notch-1 Signaling Activation and Progesterone Receptor Expression in Ectopic Lesions of Women With Endometriosis. J. Endocr. Soc..

[103] Tranguch S., Cheung-Flynn J., Daikoku T., Prapapanich V., Cox M.B., Xie H., Wang H., Das S.K., Smith D.F., Dey S.K. (2005). Cochaperone immunophilin FKBP52 is critical to uterine receptivity for embryo implantation. Proc. Natl. Acad. Sci. USA.

[104] Tranguch S., Wang H., Daikoku T., Xie H., Smith D.F., Dey S.K. (2007). FKBP52 deficiency-conferred uterine progesterone resistance is genetic background and pregnancy stage specific. J. Clin. Investig..

[105] Hirota Y., Tranguch S., Daikoku T., Hasegawa A., Osuga Y., Taketani Y., Dey S.K. (2008). Deficiency of immunophilin FKBP52 promotes endometriosis. Am. J. Pathol..

[106] Yang H., Zhou Y., Edelshain B., Schatz F., Lockwood C.J., Taylor H.S. (2012). FKBP4 is regulated by HOXA10 during decidualization and in endometriosis. Reproduction.

[107] Joshi N.R., Miyadahira E.H., Afshar Y., Jeong J.W., Young S.L., Lessey B.A., Serafini P.C., Fazleabas A.T. (2017). Progesterone Resistance in Endometriosis Is Modulated by the Altered Expression of MicroRNA-29c and FKBP4. J. Clin. Endocrinol. Metab..

[108] Jackson K.S., Brudney A., Hastings J.M., Mavrogianis P.A., Kim J.J., Fazleabas A.T. (2007). The altered distribution of the steroid hormone receptors and the chaperone immunophilin FKBP52 in a baboon model of endometriosis is associated with progesterone resistance during the window of uterine receptivity. Reprod. Sci..

[109] Gonzalez-Ramos R., Defrere S., Devoto L. (2012). Nuclear factor-kappaB: A main regulator of inflammation and cell survival in endometriosis pathophysiology. Fertil. Steril..

[110] Gonzalez-Ramos R., Rocco J., Rojas C., Sovino H., Poch A., Kohen P., Alvarado-Diaz C., Devoto L. (2012). Physiologic activation of nuclear factor kappa-B in the endometrium during the menstrual cycle is altered in endometriosis patients. Fertil. Steril..

[111] Kim B.G., Yoo J.Y., Kim T.H., Shin J.H., Langenheim J.F., Ferguson S.D., Fazleabas A.T., Young S.L., Lessey B.A., Jeong J.W. (2015). Aberrant activation of signal transducer and activator of transcription-3 (STAT3) signaling in endometriosis. Hum. Reprod..

[112] Sekulovski N., Whorton A.E., Tanaka T., Hirota Y., Shi M., MacLean J.A., de Mola J.R.L., Groesch K., Diaz-Sylvester P., Wilson T. (2020). Niclosamide suppresses macrophage-induced inflammation in endometriosisdagger. Biol. Reprod..

[113] Sekulovski N., Whorton A.E., Shi M., MacLean J.A., Hayashi K. (2019). Endometriotic inflammatory microenvironment induced by macrophages can be targeted by niclosamidedagger. Biol. Reprod..

[114] Lee J.H., Kim T.H., Oh S.J., Yoo J.Y., Akira S., Ku B.J., Lydon J.P., Jeong J.W. (2013). Signal transducer and activator of transcription-3 (Stat3) plays a critical role in implantation via progesterone receptor in uterus. FASEB J..

[115] Shi M., Sekulovski N., Whorton A.E., MacLean J.A., Greaves E., Hayashi K. (2021). Efficacy of niclosamide on the intra-abdominal inflammatory environment in endometriosis. FASEB J..

[116] Yoo J.Y., Jeong J.W., Fazleabas A.T., Tayade C., Young S.L., Lessey B.A. (2016). Protein Inhibitor of Activated STAT3 (PIAS3) Is Down-Regulated in Eutopic Endometrium of Women with Endometriosis. Biol. Reprod..

[117] Yoo J.Y., Kim T.H., Fazleabas A.T., Palomino W.A., Ahn S.H., Tayade C., Schammel D.P., Young S.L., Jeong J.W., Lessey B.A. (2017). KRAS Activation and over-expression of SIRT1/BCL6 Contributes to the Pathogenesis of Endometriosis and Progesterone Resistance. Sci. Rep..

[118] Arguni E., Arima M., Tsuruoka N., Sakamoto A., Hatano M., Tokuhisa T. (2006). JunD/AP-1 and STAT3 are the major enhancer molecules for high Bcl6 expression in germinal center B cells. Int. Immunol..

[119] Evans-Hoeker E., Lessey B.A., Jeong J.W., Savaris R.F., Palomino W.A., Yuan L., Schammel D.P., Young S.L. (2016). Endometrial BCL6 Overexpression in Eutopic Endometrium of Women With Endometriosis. Reprod. Sci..

[120] Di Cristofano A., Ellenson L.H. (2007). Endometrial carcinoma. Annu. Rev. Pathol..

[121] Rubel C.A., Lanz R.B., Kommagani R., Franco H.L., Lydon J.P., DeMayo F.J. (2012). Research resource: Genome-wide profiling of progesterone receptor binding in the mouse uterus. Mol. Endocrinol..

[122] Guimaraes-Young A., Neff T., Dupuy A.J., Goodheart M.J. (2016). Conditional deletion of Sox17 reveals complex effects on uterine adenogenesis and function. Dev. Biol..

[123] Hirate Y., Suzuki H., Kawasumi M., Takase H.M., Igarashi H., Naquet P., Kanai Y., Kanai-Azuma M. (2016). Mouse Sox17 haploinsufficiency leads to female subfertility due to impaired implantation. Sci. Rep..

[124] Wang X., Li X., Wang T., Wu S.P., Jeong J.W., Kim T.H., Young S.L., Lessey B.A., Lanz R.B., Lydon J.P. (2018). SOX17 regulates uterine epithelial-stromal cross-talk acting via a distal enhancer upstream of Ihh. Nat. Commun..

[125] Kim T.H., Yoo J.Y., Wang Z., Lydon J.P., Khatri S., Hawkins S.M., Leach R.E., Fazleabas A.T., Young S.L., Lessey B.A. (2015). ARID1A Is Essential for Endometrial Function during Early Pregnancy. PLoS Genet..

[126] Kim T.H., Yoo J.Y., Choi K.C., Shin J.H., Leach R.E., Fazleabas A.T., Young S.L., Lessey B.A., Yoon H.G., Jeong J.W. (2019). Loss of HDAC3 results in nonreceptive endometrium and female infertility. Sci. Transl. Med..

[127] Kim J.J., Taylor H.S., Lu Z., Ladhani O., Hastings J.M., Jackson K.S., Wu Y., Guo S.W., Fazleabas A.T. (2007). Altered expression of HOXA10 in endometriosis: Potential role in decidualization. Mol. Hum. Reprod..

[128] Lee B., Du H., Taylor H.S. (2009). Experimental murine endometriosis induces DNA methylation and altered gene expression in eutopic endometrium. Biol. Reprod..

[129] Dyson M.T., Roqueiro D., Monsivais D., Ercan C.M., Pavone M.E., Brooks D.C., Kakinuma T., Ono M., Jafari N., Dai Y. (2014). Genome-wide DNA methylation analysis predicts an epigenetic switch for GATA factor expression in endometriosis. PLoS Genet..

[130] Rahmioglu N., Montgomery G.W., Zondervan K.T. (2015). Genetics of endometriosis. Women’s Health.

[131] Bird C.C., McElin T.W., Manalo-Estrella P. (1972). The elusive adenomyosis of the uterus—Revisited. Am. J. Obstet. Gynecol..

[132] Benagiano G., Brosens I. (2006). History of adenomyosis. Best Pract. Res. Clin. Obstet. Gynaecol..

[133] Ferenczy A. (1998). Pathophysiology of adenomyosis. Hum. Reprod. Update.

[134] Stratopoulou C.A., Donnez J., Dolmans M.M. (2021). Origin and Pathogenic Mechanisms of Uterine Adenomyosis: What Is Known So Far. Reprod. Sci..

[135] Garcia-Solares J., Donnez J., Donnez O., Dolmans M.M. (2018). Pathogenesis of uterine adenomyosis: Invagination or metaplasia?. Fertil. Steril..

[136] Bulun S.E., Yildiz S., Adli M., Wei J.J. (2021). Adenomyosis pathogenesis: Insights from next-generation sequencing. Hum. Reprod. Update.

[137] Leyendecker G., Wildt L. (2011). A new concept of endometriosis and adenomyosis: Tissue injury and repair (TIAR). Horm. Mol. Biol. Clin. Investig..

[138] Batt R.E., Yeh J. (2013). Mullerianosis: Four developmental (embryonic) mullerian diseases. Reprod. Sci..

[139] Enatsu A., Harada T., Yoshida S., Iwabe T., Terakawa N. (2000). Adenomyosis in a patient with the Rokitansky-Kuster-Hauser syndrome. Fertil. Steril..

[140] Koninckx P.R., Ussia A., Adamyan L., Wattiez A., Gomel V., Martin D.C. (2019). Pathogenesis of endometriosis: The genetic/epigenetic theory. Fertil. Steril..

[141] Hashimoto A., Iriyama T., Sayama S., Nakayama T., Komatsu A., Miyauchi A., Nishii O., Nagamatsu T., Osuga Y., Fujii T. (2018). Adenomyosis and adverse perinatal outcomes: Increased risk of second trimester miscarriage, preeclampsia, and placental malposition. J. Matern. Fetal Neonatal Med..

[142] Vannuccini S., Clifton V.L., Fraser I.S., Taylor H.S., Critchley H., Giudice L.C., Petraglia F. (2016). Infertility and reproductive disorders: Impact of hormonal and inflammatory mechanisms on pregnancy outcome. Hum. Reprod. Update.

[143] Struble J., Reid S., Bedaiwy M.A. (2016). Adenomyosis: A Clinical Review of a Challenging Gynecologic Condition. J. Minim. Invasive Gynecol..

[144] Abbott J.A. (2017). Adenomyosis and Abnormal Uterine Bleeding (AUB-A)-Pathogenesis, diagnosis, and management. Best Pract. Res. Clin. Obstet. Gynaecol..

[145] Gunther R., Walker C. (2021). Adenomyosis.

[146] Vercellini P., Vigano P., Somigliana E., Daguati R., Abbiati A., Fedele L. (2006). Adenomyosis: Epidemiological factors. Best Pract. Res. Clin. Obstet. Gynaecol..

[147] Yu O., Schulze-Rath R., Grafton J., Hansen K., Scholes D., Reed S.D. (2020). Adenomyosis incidence, prevalence and treatment: United States population-based study 2006–2015. Am. J. Obstet. Gynecol..

[148] Bourdon M., Santulli P., Marcellin L., Maignien C., Maitrot-Mantelet L., Bordonne C., Plu Bureau G., Chapron C. (2021). Adenomyosis: An update regarding its diagnosis and clinical features. J. Gynecol. Obstet. Hum. Reprod..

[149] Pinzauti S., Lazzeri L., Tosti C., Centini G., Orlandini C., Luisi S., Zupi E., Exacoustos C., Petraglia F. (2015). Transvaginal sonographic features of diffuse adenomyosis in 18–30-year-old nulligravid women without endometriosis: Association with symptoms. Ultrasound Obstet. Gynecol..

[150] Chapron C., Tosti C., Marcellin L., Bourdon M., Lafay-Pillet M.C., Millischer A.E., Streuli I., Borghese B., Petraglia F., Santulli P. (2017). Relationship between the magnetic resonance imaging appearance of adenomyosis and endometriosis phenotypes. Hum. Reprod..

[151] Sharara F.I., Kheil M.H., Feki A., Rahman S., Klebanoff J.S., Ayoubi J.M., Moawad G.N. (2021). Current and Prospective Treatment of Adenomyosis. J. Clin. Med..

[152] Vannuccini S., Luisi S., Tosti C., Sorbi F., Petraglia F. (2018). Role of medical therapy in the management of uterine adenomyosis. Fertil. Steril..

[153] Matsushima T., Akira S., Fukami T., Yoneyama K., Takeshita T. (2018). Efficacy of Hormonal Therapies for Decreasing Uterine Volume in Patients with Adenomyosis. Gynecol. Minim. Invasive Ther..

[154] Grow D.R., Filer R.B. (1991). Treatment of adenomyosis with long-term GnRH analogues: A case report. Obstet. Gynecol..

[155] Nelson J.R., Corson S.L. (1993). Long-term management of adenomyosis with a gonadotropin-releasing hormone agonist: A case report. Fertil. Steril..

[156] Fedele L., Bianchi S., Raffaelli R., Portuese A., Dorta M. (1997). Treatment of adenomyosis-associated menorrhagia with a levonorgestrel-releasing intrauterine device. Fertil. Steril..

[157] Beatty M.N., Blumenthal P.D. (2009). The levonorgestrel-releasing intrauterine system: Safety, efficacy, and patient acceptability. Ther. Clin. Risk Manag..

[158] Fong Y.F., Singh K. (1999). Medical treatment of a grossly enlarged adenomyotic uterus with the levonorgestrel-releasing intrauterine system. Contraception.

[159] Shaaban O.M., Ali M.K., Sabra A.M., Abd El Aal D.E. (2015). Levonorgestrel-releasing intrauterine system versus a low-dose combined oral contraceptive for treatment of adenomyotic uteri: A randomized clinical trial. Contraception.

[160] Abbas A.M., Samy A., Atwa K., Ghoneim H.M., Lotfy M., Saber Mohammed H., Abdellah A.M., El Bahie A.M., Aboelroose A.A., El Gedawy A.M. (2020). The role of levonorgestrel intra-uterine system in the management of adenomyosis: A systematic review and meta-analysis of prospective studies. Acta Obstet. Gynecol. Scand..

[161] Radzinsky V.E., Khamoshina M.B., Nosenko E.N., Dukhin A.O., Sojunov M.A., Orazmuradov A.A., Lebedeva M.G., Orazov M.R. (2016). Treatment strategies for pelvic pain associated with adenomyosis. Gynecol. Endocrinol..

[162] Lee K.H., Kim J.K., Lee M.A., Ko Y.B., Yang J.B., Kang B.H., Yoo H.J. (2016). Relationship between uterine volume and discontinuation of treatment with levonorgestrel-releasing intrauterine devices in patients with adenomyosis. Arch. Gynecol. Obstet..

[163] Vannuccini S., Tosti C., Carmona F., Huang S.J., Chapron C., Guo S.W., Petraglia F. (2017). Pathogenesis of adenomyosis: An update on molecular mechanisms. Reprod. Biomed. Online.

[164] Nie J., Lu Y., Liu X., Guo S.W. (2009). Immunoreactivity of progesterone receptor isoform B, nuclear factor κB, and IκBα in adenomyosis. Fertil. Steril..

[165] Mehasseb M.K., Panchal R., Taylor A.H., Brown L., Bell S.C., Habiba M. (2011). Estrogen and progesterone receptor isoform distribution through the menstrual cycle in uteri with and without adenomyosis. Fertil. Steril..

[166] Bulun S.E. (2013). Uterine fibroids. N. Engl. J. Med..

[167] Stewart E.A., Laughlin-Tommaso S.K., Catherino W.H., Lalitkumar S., Gupta D., Vollenhoven B. (2016). Uterine fibroids. Nat. Rev. Dis. Primers.

[168] Islam M.S., Ciavattini A., Petraglia F., Castellucci M., Ciarmela P. (2018). Extracellular matrix in uterine leiomyoma pathogenesis: A potential target for future therapeutics. Hum. Reprod. Update.

[169] Stewart E.A., Cookson C.L., Gandolfo R.A., Schulze-Rath R. (2017). Epidemiology of uterine fibroids: A systematic review. BJOG.

[170] Baird D.D., Dunson D.B., Hill M.C., Cousins D., Schectman J.M. (2003). High cumulative incidence of uterine leiomyoma in black and white women: Ultrasound evidence. Am. J. Obstet. Gynecol..

[171] Zimmermann A., Bernuit D., Gerlinger C., Schaefers M., Geppert K. (2012). Prevalence, symptoms and management of uterine fibroids: An international internet-based survey of 21,746 women. BMC Womens Health.

[172] Giuliani E., As-Sanie S., Marsh E.E. (2020). Epidemiology and management of uterine fibroids. Int. J. Gynaecol. Obstet..

[173] Borah B.J., Laughlin-Tommaso S.K., Myers E.R., Yao X., Stewart E.A. (2016). Association Between Patient Characteristics and Treatment Procedure Among Patients With Uterine Leiomyomas. Obstet. Gynecol..

[174] Merrill R.M. (2008). Hysterectomy surveillance in the United States, 1997 through 2005. Med. Sci. Monit..

[175] Whiteman M.K., Hillis S.D., Jamieson D.J., Morrow B., Podgornik M.N., Brett K.M., Marchbanks P.A. (2008). Inpatient hysterectomy surveillance in the United States, 2000–2004. Am. J. Obstet. Gynecol..

[176] Donnez J., Dolmans M.M. (2016). Uterine fibroid management: From the present to the future. Hum. Reprod. Update.

[177] Moroni R.M., Martins W.P., Dias S.V., Vieira C.S., Ferriani R.A., Nastri C.O., Brito L.G. (2015). Combined oral contraceptive for treatment of women with uterine fibroids and abnormal uterine bleeding: A systematic review. Gynecol. Obstet. Investig..

[178] Laughlin-Tommaso S.K., Stewart E.A. (2018). Moving Toward Individualized Medicine for Uterine Leiomyomas. Obstet. Gynecol..

[179] American College of Obstetricians and Gynecologists (2008). ACOG practice bulletin. Alternatives to hysterectomy in the management of leiomyomas. Obstet. Gynecol..

[180] Lethaby A., Vollenhoven B., Sowter M. (2001). Pre-operative GnRH analogue therapy before hysterectomy or myomectomy for uterine fibroids. Cochrane Database Syst. Rev..

[181] Ishikawa H., Ishi K., Serna V.A., Kakazu R., Bulun S.E., Kurita T. (2010). Progesterone is essential for maintenance and growth of uterine leiomyoma. Endocrinology.

[182] Qiang W., Liu Z., Serna V.A., Druschitz S.A., Liu Y., Espona-Fiedler M., Wei J.J., Kurita T. (2014). Down-regulation of miR-29b is essential for pathogenesis of uterine leiomyoma. Endocrinology.

[183] Kawaguchi K., Fujii S., Konishi I., Nanbu Y., Nonogaki H., Mori T. (1989). Mitotic activity in uterine leiomyomas during the menstrual cycle. Am. J. Obstet. Gynecol..

[184] Lamminen S., Rantala I., Helin H., Rorarius M., Tuimala R. (1992). Proliferative activity of human uterine leiomyoma cells as measured by automatic image analysis. Gynecol. Obstet. Investig..

[185] Palomba S., Sena T., Morelli M., Noia R., Zullo F., Mastrantonio P. (2002). Effect of different doses of progestin on uterine leiomyomas in postmenopausal women. Eur. J. Obstet. Gynecol. Reprod. Biol..

[186] Carr B.R., Marshburn P.B., Weatherall P.T., Bradshaw K.D., Breslau N.A., Byrd W., Roark M., Steinkampf M.P. (1993). An evaluation of the effect of gonadotropin-releasing hormone analogs and medroxyprogesterone acetate on uterine leiomyomata volume by magnetic resonance imaging: A prospective, randomized, double blind, placebo-controlled, crossover trial. J. Clin. Endocrinol. Metab..

[187] Friedman A.J., Daly M., Juneau-Norcross M., Rein M.S., Fine C., Gleason R., Leboff M. (1993). A prospective, randomized trial of gonadotropin-releasing hormone agonist plus estrogen-progestin or progestin “add-back” regimens for women with leiomyomata uteri. J. Clin. Endocrinol. Metab..

[188] Stewart E.A., Friedman A.J., Peck K., Nowak R.A. (1994). Relative overexpression of collagen type I and collagen type III messenger ribonucleic acids by uterine leiomyomas during the proliferative phase of the menstrual cycle. J. Clin. Endocrinol. Metab..

[189] Ohara N. (2009). Sex steroidal modulation of collagen metabolism in uterine leiomyomas. Clin. Exp. Obstet. Gynecol..

[190] Islam M.S., Catherino W.H., Protic O., Janjusevic M., Gray P.C., Giannubilo S.R., Ciavattini A., Lamanna P., Tranquilli A.L., Petraglia F. (2014). Role of activin-A and myostatin and their signaling pathway in human myometrial and leiomyoma cell function. J. Clin. Endocrinol. Metab..

[191] Joseph D.S., Malik M., Nurudeen S., Catherino W.H. (2010). Myometrial cells undergo fibrotic transformation under the influence of transforming growth factor beta-3. Fertil. Steril..

[192] Wang Y., Feng G., Wang J., Zhou Y., Liu Y., Shi Y., Zhu Y., Lin W., Xu Y., Li Z. (2015). Differential effects of tumor necrosis factor-alpha on matrix metalloproteinase-2 expression in human myometrial and uterine leiomyoma smooth muscle cells. Hum. Reprod..

[193] Makinen N., Mehine M., Tolvanen J., Kaasinen E., Li Y., Lehtonen H.J., Gentile M., Yan J., Enge M., Taipale M. (2011). MED12, the mediator complex subunit 12 gene, is mutated at high frequency in uterine leiomyomas. Science.

[194] Mehine M., Makinen N., Heinonen H.R., Aaltonen L.A., Vahteristo P. (2014). Genomics of uterine leiomyomas: Insights from high-throughput sequencing. Fertil. Steril..

[195] Bertsch E., Qiang W., Zhang Q., Espona-Fiedler M., Druschitz S., Liu Y., Mittal K., Kong B., Kurita T., Wei J.J. (2014). MED12 and HMGA2 mutations: Two independent genetic events in uterine leiomyoma and leiomyosarcoma. Mod. Pathol..

[196] Sandberg A.A. (2005). Updates on the cytogenetics and molecular genetics of bone and soft tissue tumors: Leiomyoma. Cancer Genet. Cytogenet..

[197] Markowski D.N., Bartnitzke S., Loning T., Drieschner N., Helmke B.M., Bullerdiek J. (2012). MED12 mutations in uterine fibroids—Their relationship to cytogenetic subgroups. Int. J. Cancer.

[198] Mehine M., Kaasinen E., Makinen N., Katainen R., Kampjarvi K., Pitkanen E., Heinonen H.R., Butzow R., Kilpivaara O., Kuosmanen A. (2013). Characterization of uterine leiomyomas by whole-genome sequencing. N. Engl. J. Med..

[199] Mehine M., Kaasinen E., Heinonen H.R., Makinen N., Kampjarvi K., Sarvilinna N., Aavikko M., Vaharautio A., Pasanen A., Butzow R. (2016). Integrated data analysis reveals uterine leiomyoma subtypes with distinct driver pathways and biomarkers. Proc. Natl. Acad. Sci. USA.

[200] Vanharanta S., Pollard P.J., Lehtonen H.J., Laiho P., Sjoberg J., Leminen A., Aittomaki K., Arola J., Kruhoffer M., Orntoft T.F. (2006). Distinct expression profile in fumarate-hydratase-deficient uterine fibroids. Hum. Mol. Genet..

[201] Wu X., Serna V.A., Thomas J., Qiang W., Blumenfeld M.L., Kurita T. (2017). Subtype-Specific Tumor-Associated Fibroblasts Contribute to the Pathogenesis of Uterine Leiomyoma. Cancer Res..

[202] Serna V.A., Wu X., Qiang W., Thomas J., Blumenfeld M.L., Kurita T. (2018). Cellular kinetics of MED12-mutant uterine leiomyoma growth and regression in vivo. Endocr. Relat. Cancer.

[203] Ikhena D.E., Liu S., Kujawa S., Esencan E., Coon J.S., Robins J., Bulun S.E., Yin P. (2018). RANKL/RANK Pathway and Its Inhibitor RANK-Fc in Uterine Leiomyoma Growth. J. Clin. Endocrinol. Metab..

[204] Liu S., Yin P., Kujawa S.A., Coon J.S., Okeigwe I., Bulun S.E. (2019). Progesterone receptor integrates the effects of mutated MED12 and altered DNA methylation to stimulate RANKL expression and stem cell proliferation in uterine leiomyoma. Oncogene.

[205] Liu S., Yin P., Xu J., Dotts A.J., Kujawa S.A., Coon V.J., Zhao H., Dai Y., Bulun S.E. (2021). Progesterone receptor-DNA methylation crosstalk regulates depletion of uterine leiomyoma stem cells: A potential therapeutic target. Stem Cell Rep..

[206] El Sabeh M., Saha S.K., Afrin S., Islam M.S., Borahay M.A. (2021). Wnt/beta-catenin signaling pathway in uterine leiomyoma: Role in tumor biology and targeting opportunities. Mol. Cell Biochem..

[207] Ono M., Yin P., Navarro A., Moravek M.B., Coon J.S., Druschitz S.A., Serna V.A., Qiang W., Brooks D.C., Malpani S.S. (2013). Paracrine activation of WNT/beta-catenin pathway in uterine leiomyoma stem cells promotes tumor growth. Proc. Natl. Acad. Sci. USA.

[208] Ono M., Yin P., Navarro A., Moravek M.B., Coon V.J., Druschitz S.A., Gottardi C.J., Bulun S.E. (2014). Inhibition of canonical WNT signaling attenuates human leiomyoma cell growth. Fertil. Steril..

[209] Liu S., Yin P., Dotts A.J., Kujawa S.A., Coon V.J., Wei J.J., Chakravarti D., Bulun S.E. (2020). Activation of protein kinase B by WNT4 as a regulator of uterine leiomyoma stem cell function. Fertil. Steril..

[210] Kim S., Xu X., Hecht A., Boyer T.G. (2006). Mediator is a transducer of Wnt/beta-catenin signaling. J. Biol. Chem..

[211] El Andaloussi A., Al-Hendy A., Ismail N., Boyer T.G., Halder S.K. (2020). Introduction of Somatic Mutation in MED12 Induces Wnt4/beta-Catenin and Disrupts Autophagy in Human Uterine Myometrial Cell. Reprod. Sci..

[212] Corachan A., Trejo M.G., Carbajo-Garcia M.C., Monleon J., Escrig J., Faus A., Pellicer A., Cervello I., Ferrero H. (2021). Vitamin D as an effective treatment in human uterine leiomyomas independent of mediator complex subunit 12 mutation. Fertil. Steril..

[213] Fauser B.C., Tarlatzis B.C., Rebar R.W., Legro R.S., Balen A.H., Lobo R., Carmina E., Chang J., Yildiz B.O., Laven J.S. (2012). Consensus on women’s health aspects of polycystic ovary syndrome (PCOS): The Amsterdam ESHRE/ASRM-Sponsored 3rd PCOS Consensus Workshop Group. Fertil. Steril..

[214] Norman R.J., Dewailly D., Legro R.S., Hickey T.E. (2007). Polycystic ovary syndrome. Lancet.

[215] Moran L.J., Hutchison S.K., Norman R.J., Teede H.J. (2011). Lifestyle changes in women with polycystic ovary syndrome. Cochrane Database Syst. Rev..

[216] Chakraborty P., Goswami S.K., Rajani S., Sharma S., Kabir S.N., Chakravarty B., Jana K. (2013). Recurrent pregnancy loss in polycystic ovary syndrome: Role of hyperhomocysteinemia and insulin resistance. PLoS ONE.

[217] Homburg R. (2004). Management of infertility and prevention of ovarian hyperstimulation in women with polycystic ovary syndrome. Best Pract. Res. Clin. Obstet. Gynaecol..

[218] Sun Y.F., Zhang J., Xu Y.M., Cao Z.Y., Wang Y.Z., Hao G.M., Gao B.L. (2020). High BMI and Insulin Resistance Are Risk Factors for Spontaneous Abortion in Patients With Polycystic Ovary Syndrome Undergoing Assisted Reproductive Treatment: A Systematic Review and Meta-Analysis. Front. Endocrinol..

[219] Scicchitano P., Dentamaro I., Carbonara R., Bulzis G., Dachille A., Caputo P., Riccardi R., Locorotondo M., Mandurino C., Matteo Ciccone M. (2012). Cardiovascular Risk in Women With PCOS. Int. J. Endocrinol. Metab..

[220] Wild R.A., Carmina E., Diamanti-Kandarakis E., Dokras A., Escobar-Morreale H.F., Futterweit W., Lobo R., Norman R.J., Talbott E., Dumesic D.A. (2010). Assessment of cardiovascular risk and prevention of cardiovascular disease in women with the polycystic ovary syndrome: A consensus statement by the Androgen Excess and Polycystic Ovary Syndrome (AE-PCOS) Society. J. Clin. Endocrinol. Metab..

[221] Dumesic D.A., Lobo R.A. (2013). Cancer risk and PCOS. Steroids.

[222] Witchel S.F., Oberfield S.E., Pena A.S. (2019). Polycystic Ovary Syndrome: Pathophysiology, Presentation, and Treatment With Emphasis on Adolescent Girls. J. Endocr. Soc..

[223] Pastor C.L., Griffin-Korf M.L., Aloi J.A., Evans W.S., Marshall J.C. (1998). Polycystic ovary syndrome: Evidence for reduced sensitivity of the gonadotropin-releasing hormone pulse generator to inhibition by estradiol and progesterone. J. Clin. Endocrinol. Metab..

[224] Hayes M.G., Urbanek M., Ehrmann D.A., Armstrong L.L., Lee J.Y., Sisk R., Karaderi T., Barber T.M., McCarthy M.I., Franks S. (2015). Genome-wide association of polycystic ovary syndrome implicates alterations in gonadotropin secretion in European ancestry populations. Nat. Commun..

[225] Diamanti-Kandarakis E., Dunaif A. (2012). Insulin resistance and the polycystic ovary syndrome revisited: An update on mechanisms and implications. Endocr. Rev..

[226] DeVane G.W., Czekala N.M., Judd H.L., Yen S.S. (1975). Circulating gonadotropins, estrogens, and androgens in polycystic ovarian disease. Am. J. Obstet. Gynecol..

[227] Baird D.T., Corker C.S., Davidson D.W., Hunter W.M., Michie E.A., Van Look P.F. (1977). Pituitary-ovarian relationships in polycystic ovary syndrome. J. Clin. Endocrinol. Metab..

[228] Franks S., Stark J., Hardy K. (2008). Follicle dynamics and anovulation in polycystic ovary syndrome. Hum. Reprod. Update.

[229] Escobar-Morreale H.F. (2018). Polycystic ovary syndrome: Definition, aetiology, diagnosis and treatment. Nat. Rev. Endocrinol..

[230] Vrbikova J., Cibula D. (2005). Combined oral contraceptives in the treatment of polycystic ovary syndrome. Hum. Reprod. Update.

[231] Wild S., Pierpoint T., Jacobs H., McKeigue P. (2000). Long-term consequences of polycystic ovary syndrome: Results of a 31 year follow-up study. Hum. Fertil..

[232] Fearnley E.J., Marquart L., Spurdle A.B., Weinstein P., Webb P.M., Australian Ovarian Cancer Study Group, The Australian National Endometrial Cancer Study Group (2010). Polycystic ovary syndrome increases the risk of endometrial cancer in women aged less than 50 years: An Australian case-control study. Cancer Causes Control.

[233] Hardiman P., Pillay O.C., Atiomo W. (2003). Polycystic ovary syndrome and endometrial carcinoma. Lancet.

[234] Goodarzi M.O., Dumesic D.A., Chazenbalk G., Azziz R. (2011). Polycystic ovary syndrome: Etiology, pathogenesis and diagnosis. Nat. Rev. Endocrinol..

[235] Perez-Medina T., Bajo J., Folgueira G., Haya J., Ortega P. (1999). Atypical endometrial hyperplasia treatment with progestogens and gonadotropin-releasing hormone analogues: Long-term follow-up. Gynecol. Oncol..

[236] Piltonen T.T., Chen J.C., Khatun M., Kangasniemi M., Liakka A., Spitzer T., Tran N., Huddleston H., Irwin J.C., Giudice L.C. (2015). Endometrial stromal fibroblasts from women with polycystic ovary syndrome have impaired progesterone-mediated decidualization, aberrant cytokine profiles and promote enhanced immune cell migration in vitro. Hum. Reprod..

[237] Quezada S., Avellaira C., Johnson M.C., Gabler F., Fuentes A., Vega M. (2006). Evaluation of steroid receptors, coregulators, and molecules associated with uterine receptivity in secretory endometria from untreated women with polycystic ovary syndrome. Fertil. Steril..

[238] Hu M., Li J., Zhang Y., Li X., Brannstrom M., Shao L.R., Billig H. (2018). Endometrial progesterone receptor isoforms in women with polycystic ovary syndrome. Am. J. Transl. Res..

[239] Lashen H. (2010). Role of metformin in the management of polycystic ovary syndrome. Ther. Adv. Endocrinol. Metab..

[240] Johnson N.P. (2014). Metformin use in women with polycystic ovary syndrome. Ann. Transl. Med..

[241] Takemura Y., Osuga Y., Yoshino O., Hasegawa A., Hirata T., Hirota Y., Nose E., Morimoto C., Harada M., Koga K. (2007). Metformin suppresses interleukin (IL)-1beta-induced IL-8 production, aromatase activation, and proliferation of endometriotic stromal cells. J. Clin. Endocrinol. Metab..

[242] Xie Y., Wang Y.L., Yu L., Hu Q., Ji L., Zhang Y., Liao Q.P. (2011). Metformin promotes progesterone receptor expression via inhibition of mammalian target of rapamycin (mTOR) in endometrial cancer cells. J. Steroid Biochem. Mol. Biol..

[243] Shen Z.Q., Zhu H.T., Lin J.F. (2008). Reverse of progestin-resistant atypical endometrial hyperplasia by metformin and oral contraceptives. Obstet. Gynecol..

[244] Session D.R., Kalli K.R., Tummon I.S., Damario M.A., Dumesic D.A. (2003). Treatment of atypical endometrial hyperplasia with an insulin-sensitizing agent. Gynecol. Endocrinol..

[245] Stochino-Loi E., Major A.L., Gillon T.E.R., Ayoubi J.M., Feki A., Bouquet de Joliniere J. (2021). Metformin, the Rise of a New Medical Therapy for Endometriosis? A Systematic Review of the Literature. Front. Med..

[246] Kim J.J., Chapman-Davis E. (2010). Role of progesterone in endometrial cancer. Semin. Reprod. Med..

[247] Montgomery B.E., Daum G.S., Dunton C.J. (2004). Endometrial hyperplasia: A review. Obstet. Gynecol. Surv..

[248] Singh G., Puckett Y. (2021). Endometrial Hyperplasia.

[249] Urick M.E., Bell D.W. (2019). Clinical actionability of molecular targets in endometrial cancer. Nat. Rev. Cancer.

[250] Emons G., Beckmann M.W., Schmidt D., Mallmann P., Uterus commission of the Gynecological Oncology Working Group (2015). New WHO Classification of Endometrial Hyperplasias. Geburtshilfe Frauenheilkd.

[251] Li L., Yue P., Song Q., Yen T.T., Asaka S., Wang T.L., Beavis A.L., Fader A.N., Jiao Y., Yuan G. (2021). Genome-wide mutation analysis in precancerous lesions of endometrial carcinoma. J. Pathol..

[252] Mencaglia L., Valle R.F., Perino A., Gilardi G. (1990). Endometrial carcinoma and its precursors: Early detection and treatment. Int. J. Gynaecol. Obstet..

[253] Linkov F., Edwards R., Balk J., Yurkovetsky Z., Stadterman B., Lokshin A., Taioli E. (2008). Endometrial hyperplasia, endometrial cancer and prevention: Gaps in existing research of modifiable risk factors. Eur. J. Cancer.

[254] Pandey J., Yonder S. (2021). Premalignant Lesions of The Endometrium.

[255] Kurman R.J., Kaminski P.F., Norris H.J. (1985). The behavior of endometrial hyperplasia. A long-term study of “untreated” hyperplasia in 170 patients. Cancer.

[256] Lacey J.V., Mutter G.L., Nucci M.R., Ronnett B.M., Ioffe O.B., Rush B.B., Glass A.G., Richesson D.A., Chatterjee N., Langholz B. (2008). Risk of subsequent endometrial carcinoma associated with endometrial intraepithelial neoplasia classification of endometrial biopsies. Cancer.

[257] Kaku T., Tsukamoto N., Hachisuga T., Tsuruchi N., Sakai K., Hirakawa T., Amada S., Saito T., Kamura T., Nakano H. (1996). Endometrial carcinoma associated with hyperplasia. Gynecol. Oncol..

[258] Trimble C.L., Kauderer J., Zaino R., Silverberg S., Lim P.C., Burke J.J., Alberts D., Curtin J. (2006). Concurrent endometrial carcinoma in women with a biopsy diagnosis of atypical endometrial hyperplasia: A Gynecologic Oncology Group study. Cancer.

[259] Gucer F., Reich O., Tamussino K., Bader A.A., Pieber D., Scholl W., Haas J., Petru E. (1998). Concomitant endometrial hyperplasia in patients with endometrial carcinoma. Gynecol. Oncol..

[260] Jarboe E.A., Mutter G.L. (2010). Endometrial intraepithelial neoplasia. Semin. Diagn. Pathol..

[261] Huvila J., Pors J., Thompson E.F., Gilks C.B. (2021). Endometrial carcinoma: Molecular subtypes, precursors and the role of pathology in early diagnosis. J. Pathol..

[262] Carugno J., Marbin S.J., Lagan A.A., Vitale S.G., Alonso L., Di Spezio Sardo A., Haimovich S. (2021). New development on hysteroscopy for endometrial cancer diagnosis: State of the art. Minerva Med..

[263] Yen T.T., Wang T.L., Fader A.N., Shih I.M., Gaillard S. (2020). Molecular Classification and Emerging Targeted Therapy in Endometrial Cancer. Int. J. Gynecol. Pathol..

[264] Albertini A.F., Devouassoux-Shisheboran M., Genestie C. (2012). Pathology of endometrioid carcinoma. Bull. Cancer.

[265] Setiawan V.W., Yang H.P., Pike M.C., McCann S.E., Yu H., Xiang Y.B., Wolk A., Wentzensen N., Weiss N.S., Webb P.M. (2013). Type I and II endometrial cancers: Have they different risk factors?. J. Clin. Oncol..

[266] Kandoth C., Schultz N., Cherniack A.D., Akbani R., Liu Y., Shen H., Robertson A.G., Pashtan I., Shen R., Cancer Genome Atlas Research Network (2013). Integrated genomic characterization of endometrial carcinoma. Nature.

[267] Salvesen H.B., Stefansson I., Kretzschmar E.I., Gruber P., MacDonald N.D., Ryan A., Jacobs I.J., Akslen L.A., Das S. (2004). Significance of PTEN alterations in endometrial carcinoma: A population-based study of mutations, promoter methylation and PTEN protein expression. Int. J. Oncol..

[268] Risinger J.I., Hayes K., Maxwell G.L., Carney M.E., Dodge R.K., Barrett J.C., Berchuck A. (1998). PTEN mutation in endometrial cancers is associated with favorable clinical and pathologic characteristics. Clin. Cancer Res..

[269] Ayhan A., Mao T.L., Suryo Rahmanto Y., Zeppernick F., Ogawa H., Wu R.C., Wang T.L., Shih Ie M. (2015). Increased proliferation in atypical hyperplasia/endometrioid intraepithelial neoplasia of the endometrium with concurrent inactivation of ARID1A and PTEN tumour suppressors. J. Pathol. Clin. Res..

[270] Joshi A., Miller C., Baker S.J., Ellenson L.H. (2015). Activated mutant p110alpha causes endometrial carcinoma in the setting of biallelic Pten deletion. Am. J. Pathol..

[271] Daikoku T., Hirota Y., Tranguch S., Joshi A.R., DeMayo F.J., Lydon J.P., Ellenson L.H., Dey S.K. (2008). Conditional loss of uterine Pten unfailingly and rapidly induces endometrial cancer in mice. Cancer Res..

[272] Kim T.H., Wang J., Lee K.Y., Franco H.L., Broaddus R.R., Lydon J.P., Jeong J.W., Demayo F.J. (2010). The Synergistic Effect of Conditional Pten Loss and Oncogenic K-ras Mutation on Endometrial Cancer Development Occurs via Decreased Progesterone Receptor Action. J. Oncol..

[273] Suryo Rahmanto Y., Shen W., Shi X., Chen X., Yu Y., Yu Z.C., Miyamoto T., Lee M.H., Singh V., Asaka R. (2020). Inactivation of Arid1a in the endometrium is associated with endometrioid tumorigenesis through transcriptional reprogramming. Nat. Commun..

[274] Wang X., Khatri S., Broaddus R., Wang Z., Hawkins S.M. (2016). Deletion of Arid1a in Reproductive Tract Mesenchymal Cells Reduces Fertility in Female Mice. Biol. Reprod..

[275] Joshi A., Wang H., Jiang G., Douglas W., Chan J.S., Korach K.S., Ellenson L.H. (2012). Endometrial tumorigenesis in Pten(+/−) mice is independent of coexistence of estrogen and estrogen receptor alpha. Am. J. Pathol..

[276] Yang S., Thiel K.W., Leslie K.K. (2011). Progesterone: The ultimate endometrial tumor suppressor. Trends Endocrinol. Metab..

[277] Committee on Gynecologic Practice (2015). The American College of Obstetricians and Gynecologists Committee Opinion no. 631. Endometrial intraepithelial neoplasia. Obstet. Gynecol..

[278] Guillon S., Popescu N., Phelippeau J., Koskas M. (2019). A systematic review and meta-analysis of prognostic factors for remission in fertility-sparing management of endometrial atypical hyperplasia and adenocarcinoma. Int. J. Gynaecol. Obstet..

[279] Trimble C.L., Method M., Leitao M., Lu K., Ioffe O., Hampton M., Higgins R., Zaino R., Mutter G.L., Society of Gynecologic Oncology Clinical Practice Committee (2012). Management of endometrial precancers. Obstet. Gynecol..

[280] Terakawa N., Kigawa J., Taketani Y., Yoshikawa H., Yajima A., Noda K., Okada H., Kato J., Yakushiji M., Tanizawa O. (1997). The behavior of endometrial hyperplasia: A prospective study. Endometrial Hyperplasia Study Group. J. Obstet. Gynaecol. Res..

[281] Gallos I.D., Shehmar M., Thangaratinam S., Papapostolou T.K., Coomarasamy A., Gupta J.K. (2010). Oral progestogens vs levonorgestrel-releasing intrauterine system for endometrial hyperplasia: A systematic review and metaanalysis. Am. J. Obstet. Gynecol..

[282] Varma R., Soneja H., Bhatia K., Ganesan R., Rollason T., Clark T.J., Gupta J.K. (2008). The effectiveness of a levonorgestrel-releasing intrauterine system (LNG-IUS) in the treatment of endometrial hyperplasia—A long-term follow-up study. Eur. J. Obstet. Gynecol. Reprod. Biol..

[283] Fleming G.F. (2015). Second-Line Therapy for Endometrial Cancer: The Need for Better Options. J. Clin. Oncol..

[284] Lacey J.V., Sherman M.E., Rush B.B., Ronnett B.M., Ioffe O.B., Duggan M.A., Glass A.G., Richesson D.A., Chatterjee N., Langholz B. (2010). Absolute risk of endometrial carcinoma during 20-year follow-up among women with endometrial hyperplasia. J. Clin. Oncol..

[285] Fazleabas A.T. (2010). Progesterone resistance in a baboon model of endometriosis. Semin. Reprod. Med..

